# FmH2ST: foundation model-based spatial transcriptomics generation from histological images

**DOI:** 10.1093/nar/gkaf865

**Published:** 2025-09-09

**Authors:** Yuequn Wang, Jun Wang, Yanyu Xu, Ning Liu, Bin Liu, Yuliang Li, Guoxian Yu

**Affiliations:** School of Software, Shandong University, Jinan 250101, Shandong, China; SDU-NTU Centre for Artificial Intelligence Research, Shandong University, Jinan 250101, Shandong, China; SDU-NTU Centre for Artificial Intelligence Research, Shandong University, Jinan 250101, Shandong, China; SDU-NTU Centre for Artificial Intelligence Research, Shandong University, Jinan 250101, Shandong, China; School of Software, Shandong University, Jinan 250101, Shandong, China; Department of Interventional Medicine and Minimally Invasive Oncology, Shandong University, Jinan 250033, Shandong, China; Department of Interventional Medicine and Minimally Invasive Oncology, Shandong University, Jinan 250033, Shandong, China; School of Software, Shandong University, Jinan 250101, Shandong, China; SDU-NTU Centre for Artificial Intelligence Research, Shandong University, Jinan 250101, Shandong, China

## Abstract

Spatial transcriptomics (ST) reveals gene expression distributions within tissues. Yet, predicting spatial gene expression from histological images still faces the challenges of limited ST data that lack prior knowledge, and insufficient capturing of inter-slice heterogeneity and intra-slice complexity. To tackle these challenges, we introduce FmH2ST, a foundation model-based method for spatial gene expression prediction. Equipped with powerful foundation models pretrained on massive images, FmH2ST employs a dual-branch framework to integrate prior knowledge from foundation model and fine-grained details from spot images. The foundation model branch employs a multilevel feature extraction strategy to obtain enriched features with slice context for capturing inter-slice heterogeneity, and a dual-graph strategy to obtain spatial and semantic enriched features for modeling intra-slice complexity. The spot-specific learning branch leverages multiscale convolutions, Transformer, and graph neural network to extract fine-grained spot features. The outputs of two branches are adaptively fused for better prediction under a collaborative branch learning strategy. Experimental results show FmH2ST outperforms state-of-the-art methods on benchmark datasets. FmH2ST can denoise the raw gene expressions, reveal cancer spatial heterogeneity and gene co-expression patterns, and support the inference of gene regulatory networks. Overall, FmH2ST is effective for predicting ST, with potential applications in clinical diagnostics and personalized treatment.

## Graphical Abstract

**Graphical Abstract:**
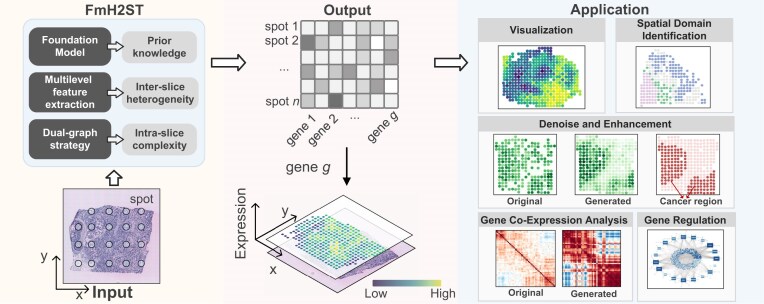

## Introduction

Histological images provide critical insights into microscopic tissue structures in clinical research and diagnosis, and their integration with gene expression profiling has become a powerful approach for investigating the structural and spatial patterns of tissues [1], advancing disease research and assessment [2]. The recent various spatial transcriptomics (ST) technologies [3, 4] enable gene expression profiling as well as retain spatial information, which have significantly enriched our insights into organ development [5], disease mechanisms [6], tumor microenvironment [7], and gene regulatory patterns [8, 9]. Particularly, ST data provide key insights for precise cancer diagnosis, uncovering the mechanisms of tumor initiation, progression, and metastasis [2, 10].

**Figure 1.**
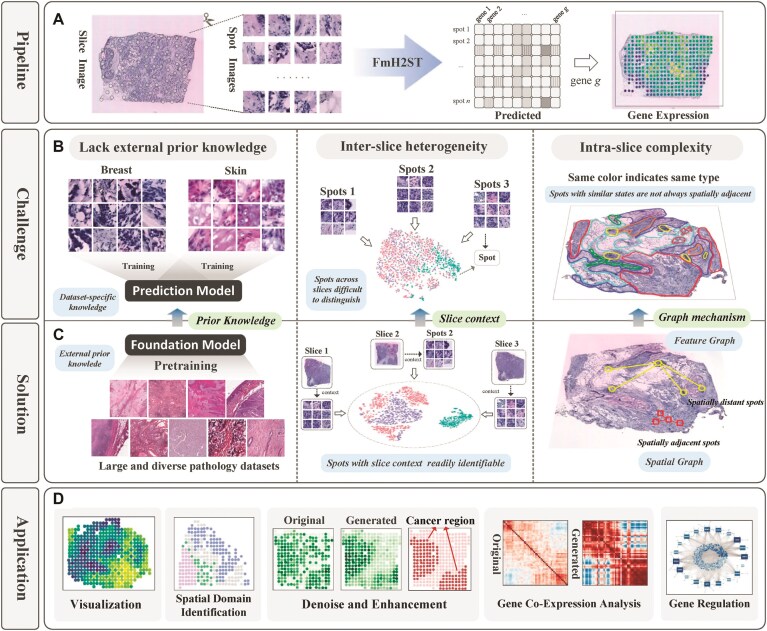
ST prediction: pipeline, challenges, solutions, and applications. (**A**) Pipeline: The workflow begins with a tissue slice image that is segmented into spot images, followed by gene expression prediction through FmH2ST, resulting in spatial gene expression maps. (**B**) Challenge: Three key limitations in current approaches include lack of external prior knowledge (models are often trained on specific tissue types), inter-slice heterogeneity (spots across different slices are difficult to distinguish), and intra-slice complexity (spots with similar types are spatially complexly distributed). (**C**) Solution: FmH2ST addresses these challenges through a foundation model pre-trained on large and diverse pathology datasets to acquire broad prior knowledge, incorporation of slice context information to identify spots across different slices, and a graph mechanism to capture complex intra-slice relationships. (**D**) Application: The predicted ST data enables multiple downstream applications including visualization, spatial domain identification, gene expression denoising and enhancement, gene co-expression analysis, and gene regulation studies.

However, the high cost and complexity of sequencing ST data have limited the widespread applications in clinical settings [11]. Fortunately, the high-resolution histological images, particularly hematoxylin and eosin (H&E) stained images, are routinely generated in clinical practice and much more cost-effective to obtain than ST data. Therefore, predicting gene expression from these histological images offers a compelling alternative to time-consuming and expensive sequencing, providing an efficient and accessible means to obtain valuable gene expression insights for clinical applications (Fig. 1A). This approach has the potential to boost clinical diagnostics and personalized treatment.

Recent studies have shown promising results in predicting gene expression from histology images. Early attempts, such as HE2RNA [12], pioneer bulk gene expression profile prediction but cannot generate spatially resolved expression. Follow-up studies utilize ST data to make spatially resolved predictions of gene expression. Among them, NSL [13], DeepSpaCE [14], and ST-Net [15] achieve spatially resolved gene expression prediction from histological images, but they only use local spot images for prediction, without exploiting spatial information. Other methods, such as HisToGene [16], Hist2ST [17], THItoGene [18], and mclSTExp [19], further leverage the spatial information. Particularly, HisToGene integrates the histological images and spatial information via the Vision Transformer [20], but it overlooks critical 2D visual information during the vectorization of patch features. Hist2ST and THItoGene leverage convolutional operations to improve the local feature extraction and model inter-spot relationships with transformers and graph neural networks (GNNs). Transformers capture the global spatial dependencies, while GNNs focus on the local neighborhood interactions. mclSTExp leverages multimodal contrastive learning to align image and gene expression features in a shared space for prediction. Different from previous methods that model inter-spot relationships, TCGN (Transformer with Convolution and Graph-Node co-embedding method) [21] focuses solely on spot images and optimizes the intra-spot feature extraction. It outperforms Hist2ST even without spatial information. The above methods focus on spot-level prediction, scstGCN [22] pushes the resolution further by reconstructing spatial expression at the single-cell level.

While these methods have made significant advancements, they still face **three limitations** (Fig. 1B). First, a common limitation of these methods is that they are typically trained on specific datasets and rely solely on internal data, **lacking external prior knowledge** and broader contextual understanding of pathological interactions. When dealing with complex or challenging tasks, the model’s predictive performance is generally constrained by available features of the specific dataset and leads to suboptimal results. Fortunately, foundation models, pre-trained on huge-scale datasets, offer a promising avenue. Their ability to extract general and rich representations [23] has been demonstrated to improve performance across various tasks [24, 25]. In particular, computational pathology foundation models [26–28], trained on diverse tissue samples, show great potential for learning contextual information about tumors, tissues, and cells [29], potentially enhancing gene expression prediction. Second, these methods have limitations in addressing **inter-slice heterogeneity**. For ST data derived from the same tissue slice, the gene expressions of individual spots generally align with the global features of the entire tissue slice [30], while exhibiting heterogeneity between different slices [31]. However, as the spots represent small regions, their features from different tissue slices are difficult to distinguish. Therefore, it is important to integrate the slice-level context, which enables the model to better differentiate spots from different slices and improves the learning of tissue context. Third, existing methods neglect the **complex intra-slice relationships**. Although methods like Hist2ST and THItoGene model intra-slice relationships via spatial graphs based on the spot spatial distance, spots with similar cell types/states may not always be spatially adjacent to each other [32]. These methods miss the biological meaningful connections between spatially distant spots, which suggests that advanced graph mechanisms need to be introduced.

In this study, we develop FmH2ST, a foundation model-based method for spatial gene expression prediction to address the three key limitations (Fig. 1C). To incorporate external prior knowledge, (i) a *dual-branch framework* is designed to fuse general priors from foundation model with fine-grained details from spot images. In the foundation model branch, two innovative strategies are adopted to enhance spot representations. (ii) A*multilevel feature extraction strategy* is introduced to model inter-slice heterogeneity by integrating features from both local spots and global tissue slices. We observe that the spot features from different tissue slices are intertwined, while the features of whole-slice images are more distinctly separable. So FmH2ST simultaneously fuses the spot- and slice-level features to enhance spot features with slice context. (iii) A *dual-graph strategy* is defined to complement the canonically used spatial graph with a learnable feature graph, and thus to model the intra-slice complexity. This feature graph is constructed based on the similarity of spot features, capturing the semantic relationships between similar but spatially distant spots. In this way, FmH2ST obtains spatial and semantic enriched spot features. Notably, the feature graph is learnable and continuously optimizes its connections based on updated image features and training objectives, enabling a more accurate reflection of the underlying relationships between spots. Besides, a spot-specific learning branch is designed to complement the foundation model branch by directly learning detailed features from spot images using multiscale convolutions, Transformer, and graph neural network. The outputs of these two branches are adaptively fused via an attention mechanism for better prediction under a collaborative branch learning strategy.

We study the effectiveness of FmH2ST through comprehensive evaluations on human breast cancer and cutaneous squamous cell carcinoma (cSCC) datasets [7, 31]. FmH2ST outperforms other state-of-the-art (SOTA) methods [15–18], and shows effectiveness across various downstream tasks (Fig. 1D). Notably, our generated gene expression profiles demonstrate effective denoising and enhancement, clearly manifesting cancer spatial heterogeneity while reliably preserving gene co-expression relationships. Additionally, its prominent performance in gene regulatory network (GRN) inference provides new perspectives and enables the study of complex gene interactions and regulatory mechanisms.

## Materials and methods

### Overview of FmH2ST

**Figure 2.**
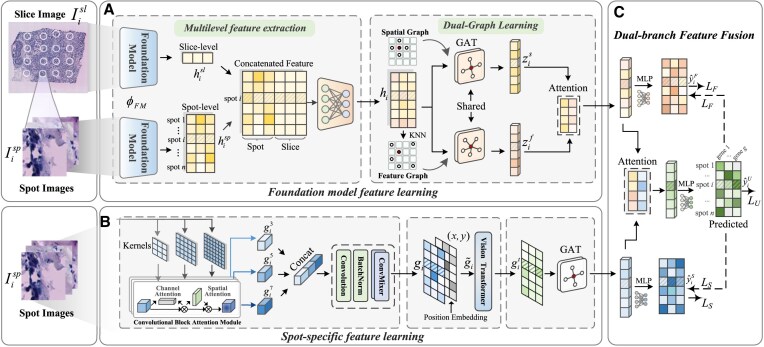
FmH2ST workflow overview. FmH2ST is a dual-branch framework for ST gene expression prediction. (**A**) The foundation model branch first uses a pre-trained foundation model (denoted as ϕ_FM_) to extract multilevel features from spot- and slice-level images for modeling inter-slice heterogeneity, and captures complex intra-slice relationships by constructing two complementary graphs: a spatial graph based on spot coordinates and a learnable feature graph based on fused features. The representations from these two graphs are fused via an attention aggregation layer. (**B**) The spot-specific branch learns fine-grained details from spot images using multiscale convolution, Transformer, and Graph Attention Network (GAT). (**C**) Features from both branches are adaptively fused through the attention mechanism. The model implements a collaborative dual-branch training strategy with a multicomponent loss (composed with *L*_F_, *L*_S_, and *L*_U_) to ensure balanced training.

The overall workflow of our proposed FmH2ST is illustrated in Fig. 2. To simultaneously incorporate prior knowledge while preserving spot specificity, FmH2ST employs a *dual-branch framework*. This framework consists of two complementary branches: the ‘foundation model feature learning branch’ (Fig. 2A), which captures generalized prior knowledge, and the ‘spot-specific feature learning branch’ (Fig. 2B), which extracts fine-grained, spot-specific features. These two branches are dynamically integrated through attention mechanisms and jointly optimized under a well-designed ‘collaborative dual-branch training’ (Fig. 2C).

Specifically, the foundation model feature branch extracts image features with valuable prior knowledge while addressing two key challenges: inter-slice heterogeneity and intra-slice complexity. To capture slice heterogeneity, we fuse *multilevel features* (spot- and slice-level) via an encoder to construct enhanced spot representations with tissue-slice context. To model complex intra-slice relationships, we employ a*dual-graph strategy* that leverages a spatial graph to capture local dependencies and a learnable feature graph to model semantic relations. These graph-specific representations are adaptively integrated through an attention mechanism.

While the foundation model branch learns enriched feature representations with prior knowledge, it may overlook fine-grained details in the spot images. To address this, FmH2ST introduces the spot-specific feature learning branch to complement the features extracted by the foundation model. This branch leverages multiscale convolutions for hierarchical spot feature extraction, a Transformer module for capturing global spatial dependencies, and GAT for local feature aggregation.

The two types of features are integrated through an attention mechanism and then projected to gene dimension through a fully connected layer to obtain predicted expression values. To mitigate potential training bias toward the foundation model branch, we employ a collaborative learning strategy inspired by previous work [33], applying loss constraints to both the final gene expression output (*L*_U_) and the intermediate branch predictions (*L*_F_ and *L*_S_).

The predicted gene expression enables multiple downstream analyses including visualization, spatial domain identification, denoise and enhancement analysis, gene co-expression analysis, and GRN inference.

### Foundation model feature learning

The foundation model branch (Fig. 2A) aims to learn feature representations with prior knowledge, while addressing the insufficient modeling of inter-slice heterogeneity and intra-slice complexity, resulting in a robust and enriched representation. To capture slice heterogeneity, we fuse *multilevel features* (spot- and slice-level) via an encoder to construct enhanced spot representations with tissue-slice context. To model complex intra-slice relationships, we employ *a dual-graph strategy* that leverages a spatial graph to capture local dependencies and a learnable feature graph to model semantic relations. After that, these graph-specific representations are adaptively integrated through an attention mechanism.

#### Slice-aware spot representation based on foundation model

In the foundation model branch, we employ a pretrained foundation model in computational pathology as the feature extractor to obtain prior pathological features. An important biological observation is that the gene expressions of individual spots generally align with the global features of the entire tissue slice [30], but exhibit heterogeneity between different slices [31]. Thus it is important to provide slice contextual information for each spot. Previous methods often model interactions between spots within slice, but overlook this inherent heterogeneity between different slices. Given that, we extract spot-level and slice-level features using a foundation model (denoted as ϕ_FM_), and fuse them to obtain enriched spot features with slice-specific context.

For spot-level features, we process the *i*-th spot image ${\bf I}_{i}^{sp}$ by ϕ_FM_ as:


(1) \begin{eqnarray*}
{{{\boldsymbol h}}}_i^{sp}= {{\phi }_{{\rm FM}}}({\bf I}_{i}^{sp})
\end{eqnarray*}

where ${{ {\boldsymbol h}}}_i^{sp}$ captures local histological patterns within spots.

To incorporate inter-slice heterogeneity, we also extract global feature ${{{\boldsymbol h}}}_i^{sl}$ by processing the corresponding slice image ${\bf I}_i^{sl}$ with ϕ_FM_:


(2) \begin{eqnarray*}
{{{\boldsymbol h}}}_i^{sl} = {{\phi }_{{\rm FM}}}({\bf I}_i^{sl})
\end{eqnarray*}

The global features capture broader tissue context and slice-specific patterns.

Finally, we concatenate the spot- and slice-level features, and process them through an multilayer perceptron (MLP) to obtain the fused feature ***h***_*i*_:


(3) \begin{eqnarray*}
{{{\boldsymbol h}}}_i = {{\rm MLP}}([{{{\boldsymbol h}}}_i^{sp} \Vert {{{\boldsymbol h}}}_i^{sl}])
\end{eqnarray*}

This multilevel feature extraction strategy captures both local and global context, providing a slice-aware spot representation that considers inter-slice heterogeneity.

#### Spatial and semantic enhanced spot representation with dual-graph learning

The representation of spot can be enhanced by considering the intra-slice relationships. In fact, a trivial way of enhancing the spot representation is to use the spatial information. However, this may lead to information loss as spots with similar states can contribute to enhance the spot representation but they may not be spatially adjacent, or even far away [32]. To model complex intra-slice relationships, we take two types of relations into account, i.e. spatial relation that reflects the effects of neighbors, and semantic relation that reflects the effects of spots with similar features. Specifically, we employ a complementary dual-graph structure: a spatial graph and a learnable feature graph. Then we introduce a Graph Attention Feature Encoder to learn both the spatial enhanced spot features and semantic enhanced spot features.

##### Spatial graph construction

To capture the local spatial dependency, we construct a spatial graph, denoted as $\mathcal {G}_s = (\mathcal {V}, \mathcal {E}_s)$. The node set $\mathcal {V}$ represents spots, and the edge set $\mathcal {E}_s$ connects each spot to its *k*_*s*_ nearest spatial neighbors. Specifically, for each spot *i* with spatial coordinates (*x*_*i*_, *y*_*i*_), we calculate its Euclidean distances to all other spots and connect it to the *k*_*s*_ nearest spots. We adopt the default *k*_*s*_ = 4 in our implementation.

##### Learnable feature graph construction

To capture semantic relationships between spatially distant yet similar spots, we construct a learnable feature graph $\mathcal {G}_f = (\mathcal {V}, \mathcal {E}_f)$ based on fused features. This feature graph complements the spatial graph and allows the model to capture nonlocal dependencies. The edge set $\mathcal {E}_f$ is constructed using a k-nearest neighbors (KNN) approach on fused features after applying principal component analysis (PCA) for dimensionality reduction. Specifically, we calculate the cosine similarity between the PCA embedding of ***h***_*i*_ and those of other spots, and connect each spot to its *k*_*f*_ nearest neighbors. We set *k*_*f*_ = 8 in our experiments through hyper-parameter analysis (detailed in Supplementary Note 1.1 and Supplementary Fig. 1).

The feature graph offers two key advantages. First, fused features integrate both global slice-level and local spot-level information, enabling the graph to connect spots based on a deeper understanding of their biological context and adapt to slice heterogeneity. Second, this graph evolves during training, allowing it to progressively capture more relevant semantic relationships between spots as the model learns better feature representations.

##### Graph attention feature encoder

To encode graph-specific feature representations, we employ GAT [34] as encoder for the spatial and feature graphs. The main advantage of GAT is the adaptive attention mechanism, which prioritizes informative neighbors during feature aggregation, allowing iterative refinement of representations. The GAT encoder learns graph-specific representations ${{\boldsymbol z}}_i^m$ (where *m* ∈ {*s*, *f*} represents the spatial and feature graphs, respectively) from the fused feature ***h***_*i*_ and its neighboring spots. This process can be formalized as:


(4) \begin{eqnarray*}
{{\boldsymbol z}}_i^m = \textrm {GAT}({{\boldsymbol h}}_i, \lbrace {{\boldsymbol h}}_j | j \in \mathcal {N}_m(i)\rbrace )
\end{eqnarray*}

where $\mathcal {N}_m(i)$ represents the neighbors of spot *i* in the spatial (or feature) graph.

Specifically, GAT uses a multihead attention mechanism. For the *k*-th attention head within graph *m*, the attention weight $e_{ij}^{m,k}$ between spots *i* and *j* is calculated as:


(5) \begin{eqnarray*}
e_{ij}^{m,k} = LeakyReLU\left({{{\boldsymbol a}}}^T[{\bf W}_h {{\boldsymbol h}}_i || {\bf W}_h {{\boldsymbol h}}_j]\right)
\end{eqnarray*}

where **W**_*h*_ is a shared linear transformation and ***a*** represents the attention mechanism, implemented as a single-layer feedforward neural network. The attention coefficient $\alpha _{ij}^{m,k}$ between spots is calculated by a softmax function:


(6) \begin{eqnarray*}
\alpha _{ij}^{m,k} =\frac{\exp \left(e_{ij}^{m,k}\right)}{\sum _{t \in \mathcal {N}_m(i)} \exp \left(e_{it}^{m,k}\right)}
\end{eqnarray*}

Subsequently, each attention head uses the attention coefficients to perform a weighted sum of the neighboring spot features. The outputs of the *K* independent attention heads are concatenated to obtain the intermediate representation ${{\boldsymbol g}}_i^m$ for the *i*-th spot:


(7) \begin{eqnarray*}
{{\boldsymbol g}}_i^m = \Big \Vert _{k=1}^K \sigma \left( \sum _{j \in \mathcal {N}_m(i)} \alpha _{ij}^{m,k} {\bf W}_h^{k} {{\boldsymbol h}}_j \right)
\end{eqnarray*}

Finally, to further fuse the representation and reduce the output dimension, we apply another graph attention layer to obtain the final graph-specific representation ${{\boldsymbol z}}_i^m$:


(8) \begin{eqnarray*}
{{\boldsymbol z}}_i^m = \sigma \left( \sum _{j \in \mathcal {N}_m(i)} \beta _{ij}^m {\bf W}_g {{\boldsymbol g}}_j^m \right)
\end{eqnarray*}

where **W**_*g*_ is a learnable weight matrix for dimensionality reduction and $\beta _{ij}^m$ represents the normalized attention coefficients in the final fusion layer, computed in the same way as $\alpha _{ij}^{m,k}$ in Equation (6).

#### Graph-specific spot representation fusion with adaptive attention aggregation

We use an attention aggregation layer to adaptively integrate spatial and feature graph representations (${{\boldsymbol z}}_i^s$ and ${{\boldsymbol z}}_i^f$) from the GAT encoder, considering that each graph contributes differently to each spot.

Specifically, for spot *i*, we first transform each graph representation ${{\boldsymbol z}}_i^m$ using a shared linear transformation (**W**_*i*_ and ***b***_*i*_). Then, we assess the importance of each graph by calculating the similarity between the transformed representation and a trainable weight vector ***q***, yielding attention scores $e_i^m$:


(9) \begin{eqnarray*}
e_i^m = \textrm {att} ({{\boldsymbol z}}_i^m) = {{\boldsymbol q}}^{T} \tanh ({\bf W}_i {{\boldsymbol z}}_i^m + {{\boldsymbol b}}_i)
\end{eqnarray*}

These scores are then normalized using softmax to obtain attention weights:


(10) \begin{eqnarray*}
\alpha _i^m=\textrm {softmax}(e_i^m) = \frac{\exp (e_i^m)}{\exp (e_i^s) + \exp (e_i^f)}
\end{eqnarray*}

Finally, the fused representation $\hat{{{\boldsymbol z}}}_i^F$ is obtained by a weighted sum of ${{\boldsymbol z}}_i^m$ using the weights:


(11) \begin{eqnarray*}
\hat{{{\boldsymbol z}}}_i^F = {\sum }_{m \in \lbrace s, f\rbrace } \alpha _i^m {{\boldsymbol z}}_i^m
\end{eqnarray*}

where $\hat{{{\boldsymbol z}}}_i^F$ represents the output features from the foundation model branch.

### Spot-specific feature learning

While the foundation model branch learns enriched feature representations with prior knowledge, it may overlook fine-grained spot-specific information in the spot images. To address this, we introduce the spot-specific feature learning branch (Fig. 2B) to complement the features extracted by the foundation model. This branch leverages multiscale convolutions for hierarchical spot feature extraction, Transformer module for capturing global spatial dependencies, and GAT for local feature aggregation.

#### Hierarchical feature extraction via multiscale convolutions

ST spot images contain multiscale information, including cells, cell clusters, and their spatial relationships. To effectively capture this information, we use multiscale convolutions combined with Convolutional Block Attention Module (CBAM) [35] to refine features through channel and spatial attention, highlighting relevant patterns.

We first employ convolution kernels of varying sizes (*n* ∈ {3, 5, 7}) to capture features at different scales (details on kernel size selection are provided in Supplementary Note 1.2 and Supplementary Fig. 2). Each convolution output is refined by a CBAM to highlight relevant features while suppressing noise:


(12) \begin{eqnarray*}
{{\boldsymbol g}}_i^{n} = CBAM_n(Conv_n({\bf I}_i^{sp}))
\end{eqnarray*}

where ${\bf I}_i^{sp}$ denotes the input spot image and ${{\boldsymbol g}}_i^n$ represents the refined feature map from kernel size *n*.

To fully integrate these multiscale features, we first perform initial fusion using 1×1 convolution and batch normalization (BN) as:


(13) \begin{eqnarray*}
{{\boldsymbol g}}_{i}^0 = BN(Conv_{1\times 1}([{{\boldsymbol g}}_i^{3} \Vert {{\boldsymbol g}}_i^{5} \Vert {{\boldsymbol g}}_i^{7}]))
\end{eqnarray*}

where ‖ denotes channel-wise concatenation. Next, we employ *L* ConvMixer blocks to capture complex feature interactions across spatial and channel dimensions. Each ConvMixer block combines depthwise convolution (DW) and pointwise convolution (PW), both followed by an activation function and BN, for spatial feature processing and cross-channel interaction, respectively. The operation of the *l*-th ConvMixer block can be described as:


(14) \begin{eqnarray*}
{{\boldsymbol g}}_i^{l} = {\rm PW}({\rm DW}({{\boldsymbol g}}_i^{l-1}) + {{\boldsymbol g}}_i^{l-1})
\end{eqnarray*}

where ${{\boldsymbol g}}_i^l$ is the output of the *l*-th block.

We then obtain the spot representation ***g***_*i*_ through downsampling to reduce feature dimensionality and flattening:


(15) \begin{eqnarray*}
{{\boldsymbol g}}_i = Flatten(Conv({{\boldsymbol g}}_i^{L}))
\end{eqnarray*}

where ${{\boldsymbol g}}_i^L$ is the output of the last ConvMixer block.

#### Spatial dependency modeling with transformer

Subsequently, we employ Transformer to capture the spatial dependencies among spots within the same slice. First, we encode the spatial information of spot *i* using learned positional embeddings ${{\boldsymbol pos}}_i^x$ and ${{\boldsymbol{pos}}}_i^y$ based on their coordinates as:


(16) \begin{eqnarray*}
\tilde{{{\boldsymbol g}}}_i = {{\boldsymbol g}}_i + {{\boldsymbol{pos}}}_i^x + {{\boldsymbol{pos}}}_i^y
\end{eqnarray*}

The Transformer module then processes these spatially aware features through multihead self-attention layers. Each attention head focuses on a specific aspect of spatial relationships between spots. For head *h*, the attention mechanism is computed as:


(17) \begin{eqnarray*}
{{\boldsymbol{head}}}_h = {Attention}({\bf Q}_h, {\bf K}_h, {\bf V}_h)
\end{eqnarray*}

(18) \begin{eqnarray*}
{Attention}({\bf Q}, {\bf K}, {\bf V}) = {\textrm {softmax}} \left( \frac{{\bf QK}^{\top }}{\sqrt{d_k}} \right) {\bf V}
\end{eqnarray*}

where **Q**_*h*_, **K**_*h*_, and **V**_*h*_ are linear projections of $\tilde{{\bf{\it g}}}_i$. The outputs of all attention heads are concatenated and then linearly transformed to produce the transformer output:


(19) \begin{eqnarray*}
{{\boldsymbol g}}_i^{t} = Concat({{\boldsymbol{head}}}_1, \cdots , {{\boldsymbol{head}}}_H){\bf W}_t
\end{eqnarray*}

where *H* is the number of heads, **W**_*t*_ is the weight matrix for aggregating the attention heads, and ${{\boldsymbol g}}_i^{t}$ is the output of the Transformer module.

#### Local feature aggregation via graph attention network

To further enhance the spot representation, we employ GAT to learn the local spatial dependency from the neighboring spots. Similar to Equation (4), for spot *i*, we model the interactions between ${{\boldsymbol g}}_i^t$ and its neighboring spots as:


(20) \begin{eqnarray*}
{\hat{{{\boldsymbol z}}}_i^S} = \textrm {GAT}({{\boldsymbol g}}_i^{t}, \lbrace {{\boldsymbol g}}_j^{t} | j \in \mathcal {N}_s(i)\rbrace )
\end{eqnarray*}

where $\mathcal {N}_s(i)$ represents the neighboring spots of spot *i* in the spatial graph, and $\hat{{{\boldsymbol z}}}_i^S$ represents the output feature from the spot-specific branch.

### Gene expression prediction

We employ an adaptive attention mechanism to fuse the enriched prior features ${\hat{{{\boldsymbol z}}}_i^F}$ from the foundation model branch and fine-grained ones ${\hat{{{\boldsymbol z}}}_i^S}$ from spot-specific branch. Subsequently, we utilize a collaborative dual-branch training strategy to prevent training bias towards foundation model branch.

#### Dual-branch feature fusion

Since different branches provide unique information for each spot, some spots may rely more on the priors from foundation model, while others benefit more from fine-grained spot-specific details. Therefore, we use an attention mechanism to dynamically fuse the features from both branches. Similar to Equations (9) and (10), for spot *i*, we first calculate attention scores to assess the importance of each branch, which are then normalized via softmax to obtain the importance weight for each branch:


(21) \begin{eqnarray*}
\hat{\alpha }_i^B = \textrm {softmax} (\textrm {att}(\hat{{{\boldsymbol z}}}_{i}^B))
\end{eqnarray*}

where *B* ∈ {*F*, *S*} indicates the foundation model branch and spot-specific branch, respectively.

Finally, the fused feature $\hat{{{\boldsymbol z}}}_i^U$ is obtained by a weighted sum of $\hat{{{\boldsymbol z}}}_i^F$ and $\hat{{{\boldsymbol z}}}_i^S$ according to their attention weights:


(22) \begin{eqnarray*}
{\hat{{{\boldsymbol z}}}_i^{U}} = \sum _{B \in \lbrace F, S\rbrace } \hat{\alpha }_i^{B} \hat{{{\boldsymbol z}}}_i^{B}
\end{eqnarray*}

#### Collaborative dual-branch training

To ensure training stability, inspired by previous work on collaborative branch learning [33], we implement a multicomponent loss function. We predict gene expression via three heads: a main prediction head on the fused features and two single-layer MLP heads on the foundation model branch and spot-specific branch, each mapping its branch features into the gene-expression space. While supervising only the fusion prediction via mean squared error (MSE) seems sufficient, this supervision approach may cause branches to rely too much on the fusion results and weaken their own learning signals. This is particularly concerning since the foundation model has been pre-trained on massive datasets, potentially leading to insufficient training of the spot-specific branch. Therefore, we generate independent gene expression predictions $\hat{{{\boldsymbol y}}}_i^U$, $\hat{{{\boldsymbol y}}}_i^{F}$, and $\hat{{{\boldsymbol y}}}_i^{S}$ using feature representations $\hat{{{\boldsymbol z}}}_i^U$, $\hat{{{\boldsymbol z}}}_i^F$, and $\hat{{{\boldsymbol z}}}_i^S$ from respective branches.

For the predictions from foundation model branch and spot-specific branch, we use a combined loss: a prediction loss (MSE between the branch’s prediction and the true gene expression *y*_*i*_), and a knowledge distillation loss (MSE between the prediction of individual branches and that of fused features). The prediction loss ensures each branch receives direct gradient signals from ground truth gene expression, while the distillation loss transfers knowledge from the fusion prediction to individual branches. The loss for branch *B* is:


(23) \begin{eqnarray*}
{L}_{{\rm B}} = \frac{1 - \alpha }{N} {\sum }_{i=1}^{N} \left( \hat{{{\boldsymbol y}}}_{i}^{B} - {{\boldsymbol y}}_{i} \right)^2 + \frac{\alpha }{N} {\sum }_{i=1}^{N} \left( \hat{{{\boldsymbol y}}}_{i}^{B} - \hat{{{\boldsymbol y}}}_{i}^U \right)^2
\end{eqnarray*}

Here, α is a hyperparameter that controls the balance between the prediction loss and the distillation loss. The fusion loss *L*_U_ is the MSE between the prediction from fused features and the true gene expression:


(24) \begin{eqnarray*}
{L}_{{\rm U}} = \frac{1}{N} \sum _{i=1}^{N} \left( \hat{{{\boldsymbol y}}}_{i}^U - {{\boldsymbol y}}_{i} \right)^2
\end{eqnarray*}

The multicomponent loss function is the sum of the above three losses:


(25) \begin{eqnarray*}
{L} = {L}_{{\rm U}} + {L}_{{\rm F}} + {L}_{{\rm S}}
\end{eqnarray*}

This multicomponent loss encourages both branches to collaboratively train and help each other, and prevents the model from relying too heavily on the features from foundation model branch. Further evaluation of this loss function is provided in Supplementary Note 1.3 and Supplementary Tables 1–3.

### Datasets and data preprocessing

We used the same ST datasets as the previous study [16–18, 21], including the human HER2-positive breast cancer (HER2+) dataset and the human cSCC dataset. Both datasets contained histology images, spatially resolved gene expression data, and their corresponding spatial coordinates. Specifically, HER2+ dataset was measured by the 10× Visium platform, initially comprising 36 tissue sections from eight patients. Following the same quality control criteria as previous studies, we retained 32 sections from seven patients that had at least 180 spots per section. The cSCC dataset was also sequenced using the 10× Visium platform, consisting of 12 tissue sections obtained from four patients, with three sections per patient.

We pre-processed the ST datasets. For the histological images, we divided each image into *N* patches based on the spot coordinates, where each patch size (*W*×*H*) corresponds to the spot diameter of 112 pixels in the ST data. For the gene expression data, we implemented a filtering and normalization pipeline. We first selected the top 1000 highly variable genes, excluding those expressed in <1000 spots across all tissue sections. We then normalized the gene feature counts within each spot via dividing them by the total feature counts of all genes, multiplied by 10^6^, and applied a natural logarithmic transformation. Model performance was evaluated using leave-one-out cross-validation. All predictions were collected for comprehensive model assessment.

### Evaluation metrics

For evaluating gene expression prediction performance, we use four primary metrics: PCC, Spearman’s rank correlation coefficient, MSE, and mean absolute error (MAE). These metrics assess the agreement between predicted gene expression values and the truths.

For evaluating spatial clustering performance, we use the Adjusted Rand Index (ARI), which measures the similarity between the predicted clustering and the known pathological annotations. The ARI ranges from −1 to 1, with a value of 1 indicating perfect agreement, 0 suggesting random agreement, and −1 representing complete disagreement. ARI is defined as:


(26) \begin{eqnarray*}
{\rm ARI }= \frac{\sum _{i,j} \binom{n_{ij}}{2} - \left[ \sum _{i} \binom{n_{i}}{2} \sum _{j} \binom{n_{j}}{2} \right] / \binom{n}{2}}{\frac{1}{2} \left[ \sum _{i} \binom{n_{i}}{2} + \sum _{j} \binom{n_{j}}{2} \right] - \left[ \sum _{i} \binom{n_{i}}{2} \sum _{j} \binom{n_{j}}{2} \right] / \binom{n}{2}}
\end{eqnarray*}

where *n* is the total number of samples, *n*_*i*_ and *n*_*j*_ denote the number of samples in the *i*-th predicted and *j*-th true clusters, respectively, and *n*_*ij*_ represents the number of samples belonging to both clusters.

### Differentially expressed genes analysis

We performed differential gene expression analysis using the function “rank_genes_groups method” in the Scanpy. The Wilcoxon rank sum test was applied to identify differentially expressed genes, and a Benjamini–Hochberg adjustment [36] was used to adjust the *P*-values. Specifically, we selected the genes with adjusted *P*-value <.05 as differentially expressed genes.

### Functional enrichment

We implemented Gene Ontology (GO) and Kyoto Encyclopedia of Genes and Genomes (KEGG) enrichment analyses on the top 50 predicted genes using the Sangerbox 2 platform [37]. We performed GO enrichment analysis for the regulons of the top 10 transcription factors (TFs) using the GSEApy package [38]. Adjusted *P*-values were obtained after the Benjamini–Hochberg adjustment [36]. Enrichment results are determined based on the condition of adjusted *P*-value <.05.

## Results

In experiments, we adopt PathoDuet [26], a pathology foundation model pretrained on millions of H&E patches, to extract features enriched with priors (detailed comparison with other foundation models is provided in Supplementary Note 1.4 and Supplementary Table 4). We evaluate the overall performance of FmH2ST compared to SOTA baselines using PCC and MSE. We validate the effectiveness of our model design by analyzing the dual-branch framework, inter-slice heterogeneity modeling, and complex intra-slice relationship modeling. Notably, FmH2ST’s predictions achieve both high accuracy and strong practical utility. First, we conduct visualization and functional analysis of top-ranked genes, highlighting their biological relevance in cancer progression. We then perform spatial domain identification through k-means clustering, demonstrating improved agreement with pathologist annotations. Further analyses illustrate FmH2ST capacity to denoise raw gene expression data and enhance cancer region delineation, uncovering cancer spatial heterogeneity. Finally, leveraging FmH2ST’s denoising capability, we investigate gene co-expression patterns and regulatory network inference, revealing that FmH2ST captures clearer co-expression relationships and biologically meaningful regulatory networks.

### FmH2ST achieves improved prediction accuracy for spatial gene expression

We compare our FmH2ST with four competitive methods, ST-Net [15], HisToGene [16], Hist2ST [17], and THItoGene [18], using leave-one-section-out cross-validation on the HER2+ (containing 32 tissue sections) and cSCC (containing 12 tissue sections) ST datasets. We evaluate the performance using multiple metrics including PCC, Spearman’s rank correlation coefficient, MSE, and MAE. Among all comparison methods, FmH2ST achieves the best results across all evaluation metrics.

**Figure 3.**
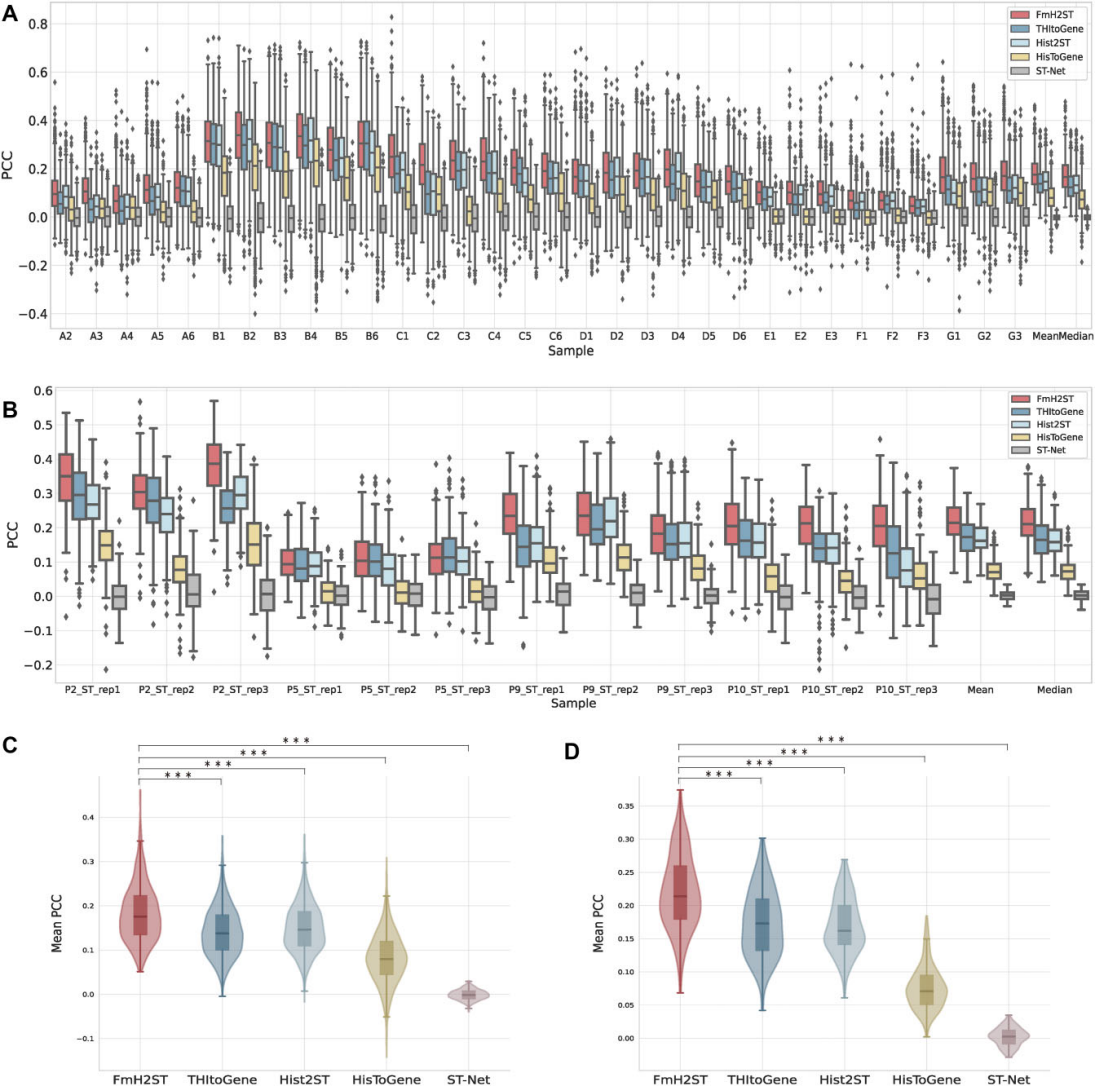
Comparison of prediction performance of different methods. (**A**, **B**) Box plots showing the distribution of PCC for different methods across tissue sections in (**A**) HER2+ dataset (32 sections) and (**B**) cSCC dataset (12 sections). (**C**, **D**) Violin plots with statistical comparison (Wilcoxon signed-rank test) of mean PCC values across all cross-validation experiments for (**C**) HER2+ dataset and (**D**) cSCC dataset. *** indicates *P*-value <.001.

The comparative analysis of PCC across both datasets is presented in Fig. 3. For both datasets, FmH2ST shows the best performance with the highest median PCC values in almost all tissue sections. Among the compared methods, ST-Net performs the worst as it only uses convolutional operations for feature extraction from spot images and does not incorporate spatial information. HisToGene shows some improvement by modeling the spatial relationships between spots, but its performance remains suboptimal. Hist2ST and THItoGene show further improvements by enhanced image feature extraction and local spot relationships modeling, but they still disregard the inter-slice heterogeneity and intra-slice complexity, and thus lose to our FmH2ST. Specifically, FmH2ST improves mean PCC by 33.8% and 27.0% over THItoGene and Hist2ST in the HER2+ dataset, and 28.4% and 32.3% in the cSCC dataset, respectively. For better statistical analysis, we perform Wilcoxon signed-rank tests on mean PCC for both datasets. As shown in Fig. 3C (HER2+) and Fig. 3D (cSCC), FmH2ST achieves highly significant statistical differences (all *P*-values <.001) compared to other methods, which confirm that the performance improvements of FmH2ST are statistically significant.

Notably, in challenging tissue sections, such as those from patient G (sections G1–G3), HisToGene, Hist2ST, and THItoGene all perform poorly. While THItoGene employs advanced modeling techniques, its performance remains inadequate, which suggests that the limited data hinders the model’s effectiveness. In contrast, by leveraging prior knowledge from the foundation model trained on large pathology datasets, FmH2ST can better handle these challenging tissue sections and significantly outperforms these compared methods, with its median PCC approaching the upper quartile of the compared methods.

While PCC provides valuable insights into linear relationships, gene expression data often follows non-normal distributions. Therefore, we also use Spearman’s rank correlation coefficient for evaluation, which is more robust to non-normal distributions and captures monotonic relationships. As shown in the Supplementary Fig. S1, FmH2ST consistently achieves the highest median Spearman correlation values in almost all tissue sections across both datasets. FmH2ST also achieves the highest mean Spearman correlation values with highly significant statistical differences (all *P*-values <.001), further validating the effectiveness of FmH2ST in capturing both linear and monotonic relationships in gene expression data.

To further validate the model performance, we analyze both MSE and MAE metrics. As shown in Supplementary Figs S2 and S3, FmH2ST achieves lower mean MSE and MAE values than other methods on both datasets. Notably, in sections A3, A4, and C2, where other methods exhibit substantially higher MSE and MAE values, FmH2ST maintains relatively low error values. This stable error distribution across tissue sections highlights the generalization ability of FmH2ST, owing to its meticulously designed technical framework.

### Effectiveness analysis of model design

To validate the effectiveness of our model design, we compare the prediction performance of different branches within our dual-branch architecture, perform t-distributed stochastic neighbor embedding (t-SNE) visualization to assess the impact of global feature integration, and visualize the learned feature graph to study its biological significance.

#### Dual-branch framework analysis

**Figure 4.**
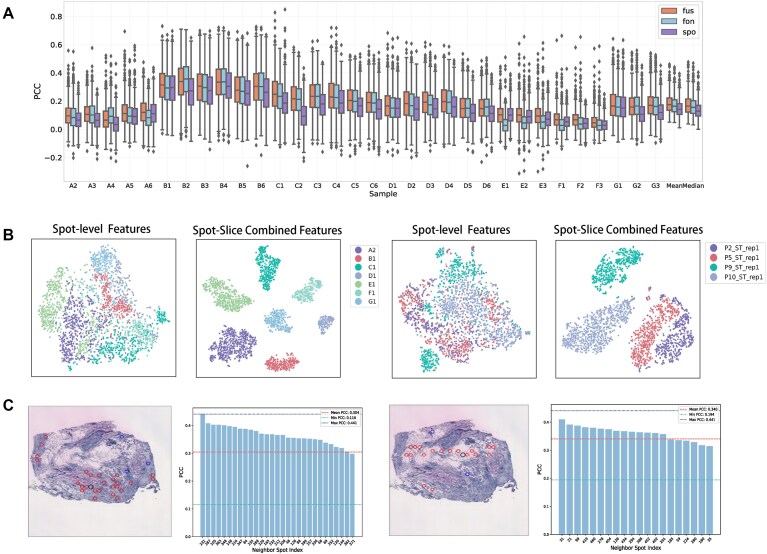
Effectiveness analysis of model design. (**A**) Performance comparison of different branches in the dual-branch architecture across tissue sections in the HER2+ dataset. The three results represent fusion prediction (fus), foundation model branch prediction (fon), and spot-specific branch prediction (spo). (**B**) Comparison of t-SNE visualization between spot-level features alone and integrated spot-level and slice-level features on HER2+ (left) and cSCC (right) datasets. (**C**) Visualization of feature graph connections and gene expression correlation on G2. The tissue slice shows feature graph connections, where the black node represents the query spot and other nodes are its neighbors in the feature graph. Red nodes represent neighbors that share the same manual annotations as the query spot. The bar plots display PCC values between the query spot and its neighbors.

To further understand our dual-branch framework, we evaluate the prediction results from three different branches: the foundation model branch, spot-specific branch, and their fusion branch on the HER2+ (Fig. 4A) and cSCC datasets (Supplementary Fig. S4). In the HER2+ dataset, we find that the fusion branch generally achieves the best performance across most tissue sections, while using either the foundation model branch or spot-specific branch alone leads to performance degradation. As to the mean PCC and median PCC across all sections, the fusion branch consistently achieves the best prediction performance.

Notably, we observe varying performances between the two branches across different tissue sections. In some sections, predictions using the foundation model branch alone outperform those using the spot-specific branch alone. However, in other sections, such as E1, E2, and E3, predictions based only on foundation model features show significantly decreased performance. This suggests that different branches are suitable for different tissue sections, validating the complementary characteristics of these two branches.

These results validate the effectiveness of FmH2ST’s dual-branch architecture, which leverages the complementary strengths of both foundation model and spot-specific features to achieve enhanced prediction performance.

#### Inter-slice heterogeneity modeling analysis

For ST data from the same tissue slice, gene expression of individual spots generally aligns with the global features of the entire slice [30], while showing heterogeneity across different slices [31]. This slice-specific heterogeneity provides valuable tissue context and influences the characteristics of individual spots.

However, our experimental analysis reveals that image features extracted from individual spots are insufficient to capture this important slice-specific information. As shown in Fig. 4B (Spot-level), when visualizing the spot features using t-SNE for selected slices from HER2+ and cSCC, spots from different slices are mixed together and difficult to differentiate, indicating that spot-level features alone carry limited information about slice-specific patterns. While methods like HisToGene use transformers to model global spatial relationships, they still rely on spot-level information and thus struggle to capture the slice context. Meanwhile, we observe that the whole-slice features demonstrate distinct separation patterns. Leveraging this biological property, we introduce slice-level global features to supplement each spot with contextual information about its tissue environment. As demonstrated in Fig. 4B (Spot-Slice), after incorporating global slice features with spot features, spots from the same slice naturally cluster together while maintaining clear boundaries between different slices.

This result justifies that our model successfully captures slice-specific patterns by incorporating global features, enabling better characterization of slice heterogeneity.

#### Intra-slice complex relationship modeling analysis

Besides capturing slice-level patterns, another key challenge is modeling meaningful relationships within slices. In complex tissue environments, spots representing similar cell types or biological states may be not always spatially adjacent [32], while existing methods solely rely on the spatial proximity to model spot relationships. To address this limitation, we design a learnable feature graph to model semantic relationships between spatially distant but similar spots, based on feature similarity. This graph dynamically updates during training as the model learns improved feature representations.

To study the biological significance of the learned feature graph, we visualize the learned connections (slice image in Fig. 4C), where the black node represents the query spot and other nodes represent feature-similar neighbors identified by FmH2ST. The visualization shows that spots connected in feature graph exhibit similar histological appearances across the slice, despite their potential spatial separation. Furthermore, each spot on the tissue slice is annotated with manual labels, which enables us to evaluate the biological relevance of these connections. Red nodes in the visualization indicate neighbors that share the same manual annotation as the query spot. A substantial portion of identified similar spots belong to the same type, confirming that our feature graph captures biologically meaningful relationships between spots.

As shown in the bar plot in Fig. 4C, to further validate the relationship between the feature graph and gene expression patterns, we analyze the PCC between the query spot and its feature graph neighbors. Results show that most connected spots have above-average PCC values, compared to the mean PCC between the query spot and all other spots (shown as pink dashed line). This indicates that the feature graph can reflect underlying gene expression correlations.

To assess the impact of each technical component, we performed an ablation study by: (i) replacing the pathology foundation model with a general vision model (ResNet18 [39]without pretrained parameters), as ResNet18 better aligns with the scale of our dataset for training from scratch and eliminates the influence of external prior knowledge; (ii) excluding slice-level global features; and (iii) removing the feature graph. As shown in Table 1, all three variants lead to increased mean MSE and decreased mean PCC compared to the complete FmH2ST, suggesting that external prior knowledge, slice-level global features, and the feature graph all contribute to improved gene expression prediction. Notably, replacing the pathology foundation model with ResNet18 leads to the most substantial performance decline, highlighting the importance of integrating prior knowledge from foundation model into FmH2ST.

**Table 1.** Ablation study for foundation model, global features and feature graph [mean MSE and mean Pearson correlation coefficient (PCC)]

	HER2-positive breast cancer (HER2+)		cSCC	
	PCC	MSE ↓	PCC	MSE ↓
W/o foundation model	0.125	0.344	0.110	0.953
W/o global features	0.158	0.278	0.206	0.647
W/o feature graph	0.172	0.260	0.202	0.640
FmH2ST	**0.184**	**0.258**	**0.220**	**0.632**

### FmH2ST provides accurate predictions for cancer-related genes

To investigate what types of genes can be more accurately predicted by FmH2ST, we analyze the correlations between observed and predicted gene expression patterns. Following the same protocols in [17, 18], we calculate correlation coefficients and corresponding *P*-values for each gene and then compute the mean −log_10_*P*-values across 32 cross-validations for all genes. These genes are ranked in descending order based on their −log_10_*P*-values, as detailed in Supplementary Table S1. We select the top four genes with the lowest mean *P*-values for comparison and visualization.

**Figure 5.**
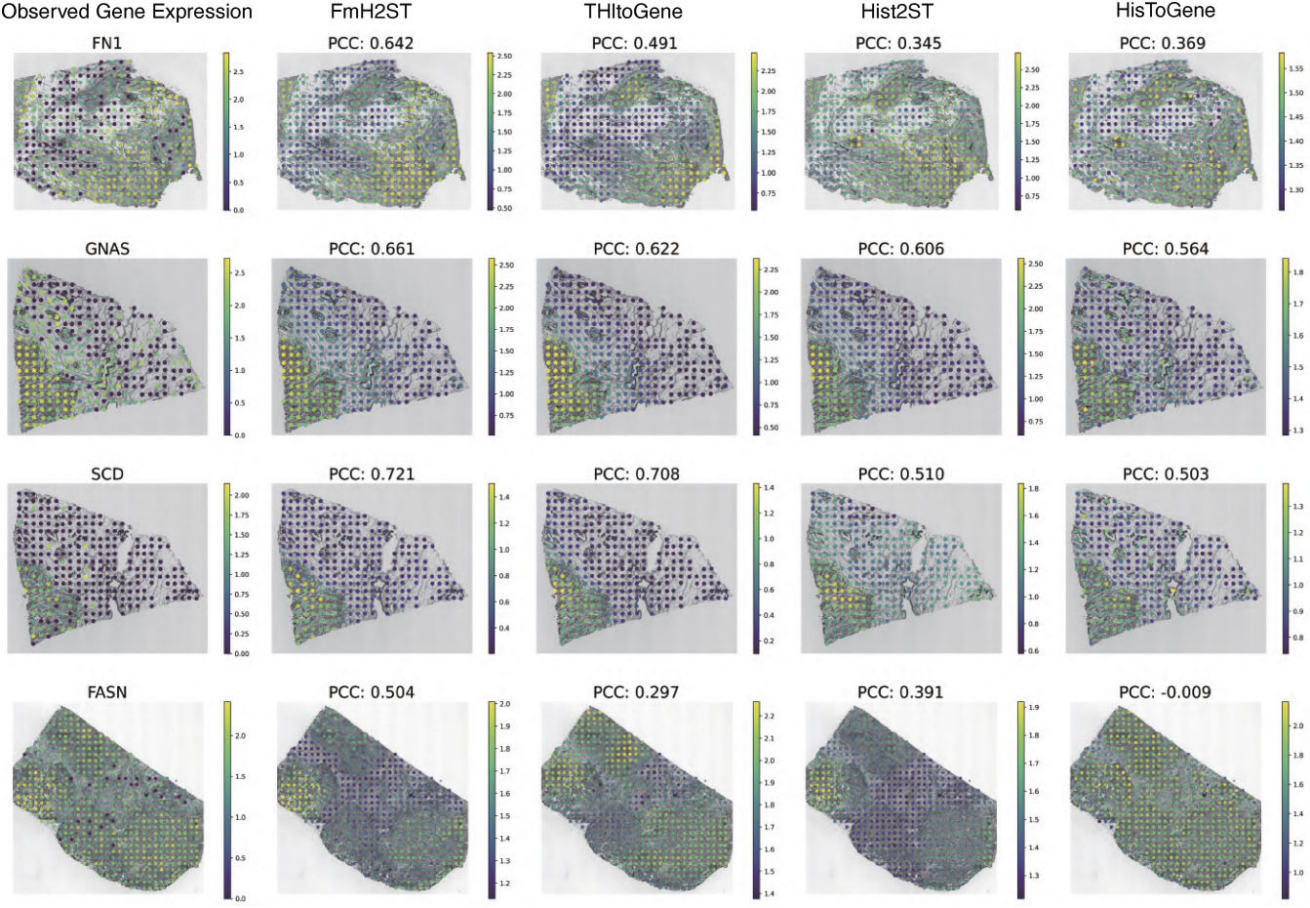
Visualization of the predicted four genes with the highest mean −log_10_*P*-values across 32 tissue sections are shown. *P*-values were calculated from correlations between predicted and observed gene expressions. For each gene, the tissue section with the lowest *P*-value is selected for visualization.

The top four genes identified by FmH2ST in the HER2+ dataset are FN1, GNAS, SCD, and FASN. We then visualize each gene expression pattern on the tissue section in which the gene shows the lowest *P*-value. As shown in Fig. 5, FmH2ST achieves the highest PCC for all four genes. Notably, these four genes are closely associated with breast cancer and recognized as potential cancer biomarkers [40–44]. Specifically, FN1 is frequently overexpressed in breast cancer and strongly linked to cancer recurrence and invasion [40]. SCD expression shows significant correlations with clinical pathological features of breast cancer, particularly high expression in HER2+ breast cancer [42]. FASN exhibits higher expression in breast cancer tissues than normal tissues, and inhibition of it affects breast cancer cell proliferation, metabolism, and migration capabilities [43]. These findings demonstrate that FmH2ST supports more accurate identification of cancer-related genes.

As illustrated in Supplementary Fig. S5 and Supplementary Table S2, we apply a similar approach to visualize four top genes (COL1A2, MSMO1, COL3A1, and NDRG1) in the cSCC dataset. FmH2ST consistently outperforms compared methods in terms of mean PCC for these genes, which are closely associated with cSCC [45–47].

**Figure 6.**
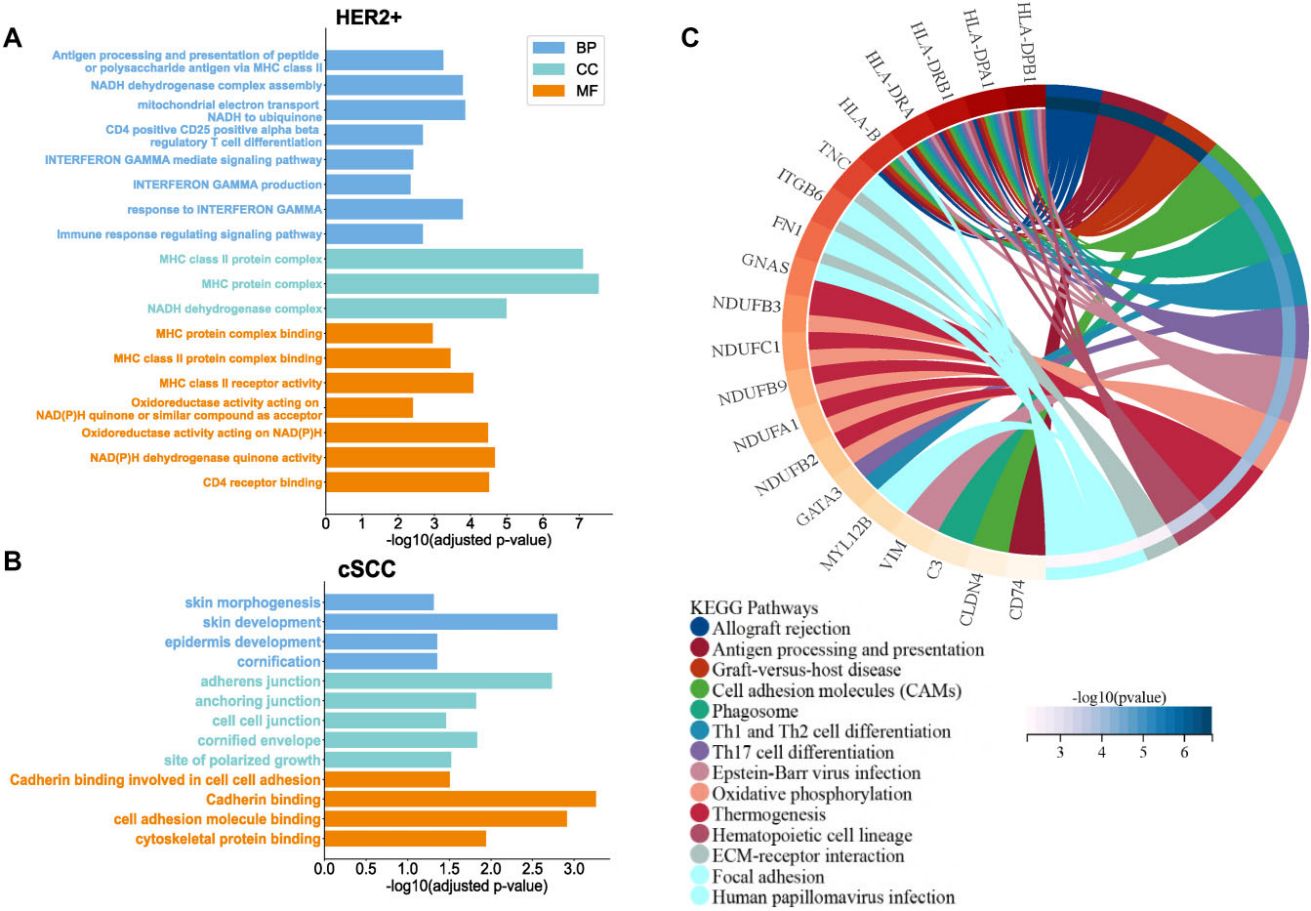
Enrichment analysis of top 50 predicted genes (selected based on mean PCC ranking among statistically significant genes with mean *P*-value <.05). (**A**) GO enrichment analysis results for HER2+ breast cancer dataset. BP, CC, and MF terms are displayed with their −log_10_ (adjusted *P*-value). (**B**) GO enrichment analysis results for cSCC dataset. (**C**) Visualization of KEGG pathway enrichment analysis for HER2+ dataset.

To further explore gene functions, similar to Hist2ST, we select genes with mean *P*-values <.05, then rank these genes based on their mean PCC and choose the top 50 genes with both high prediction accuracy and statistical significance for GO enrichment analysis. In the HER2+ dataset (Fig. 6A), GO analysis reveals enriched terms mainly related to NADH-related enzymes and major histocompatibility complex protein functions, which are closely associated with breast cancer [48–50] and consistent with previous observations [21]. GO analysis also reveals enriched terms related to immune response, such as interferon-gamma production. We further perform KEGG pathway analysis (Fig. 6C), which identifies significant enrichment in Th1 and Th2 cell differentiation pathways, consistent with the previous interferon-gamma enrichment. The imbalance of these pathways is a contributing factor to malignant tumor development [51]. KEGG analysis also shows high enrichment in cell adhesion molecules and focal adhesion pathways, which play essential roles in tumor development, invasion, and metastasis. In the cSCC dataset (Fig. 6B), GO analysis reveals pronounced enrichment in skin development and cadherin-related terms, and the reduced expression of cadherins in cSCC is closely associated with enhanced tumor invasiveness and poor prognosis [52].

Overall, these analyses demonstrate the biological relevance and validity of our predicted gene expression. The top-ranked genes identified by FmH2ST are recognized as cancer-related genes that play important roles in tumor progression and clinical prognosis. In addition, enrichment analysis of highly ranked genes reveals biological processes that are closely related to cancer development.

### FmH2ST reconstructs spatial domains from predicted gene expression

Identifying distinct spatial domains in tissue sections is crucial for understanding the spatial distribution of cells and subcellular structures [53, 54]. To explore the capability of FmH2ST to reconstruct these spatial domains, we conduct a quantitative analysis using a set of six tissue sections (B1, C1, D1, E1, F1, and G2) that are annotated by pathologists. For each tissue section, the number of clusters is set to match the number of spatial domains identified in the manual annotations. We apply k-means clustering to the gene expression predicted by three different methods: FmH2ST, THItoGene, and Hist2ST. Additionally, as a reference, we also perform k-means clustering on the observed gene expression. To evaluate the clustering performance, we calculate the ARI between the clustering results and the pathologist-annotated ground truth.

**Figure 7.**
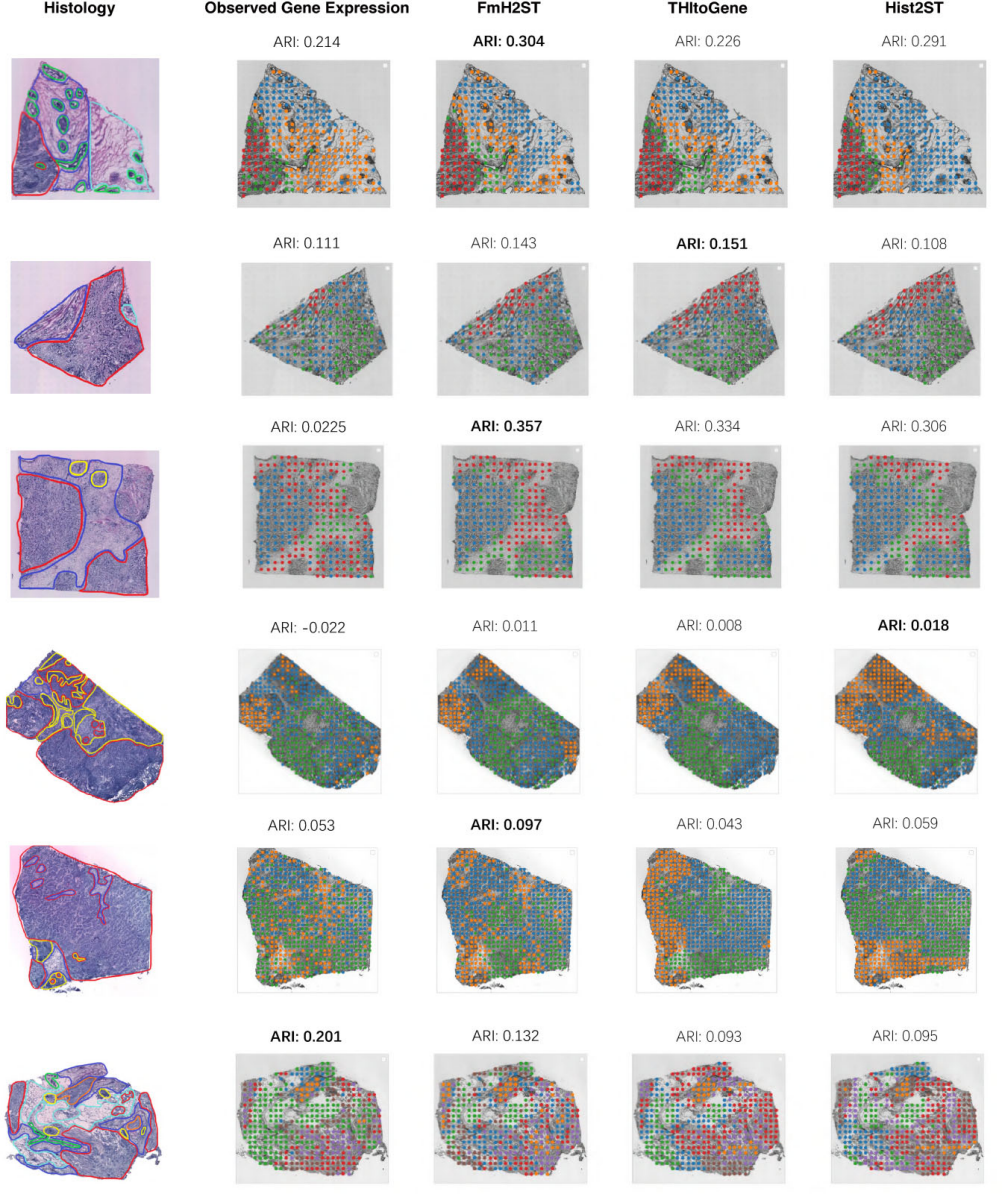
Spatial domains identified by k-means clustering using gene expressions predicted by FmH2ST, THItoGene, Hist2ST, and observed gene expression, across six tissue sections (B1, C1, D1, E1, F1, and G2). The first column shows the manual annotations of pathologists, which serve as the ground truth for calculating the ARI.

As shown in Fig. 7, FmH2ST achieves the highest mean ARI across all six tissue sections, showing a 22% improvement over THItoGene. THItoGene and Hist2ST gain comparable mean ARI scores. When examining individual tissue sections, FmH2ST achieves the best performance in four out of the six sections and ranks the second-best in the remaining two. Notably, clustering based on observed gene expression data yields the lowest average ARI among all methods, possibly due to inherent noise in sequencing data and the potential denoising effect of prediction methods that incorporate histological image information.

### FmH2ST achieves denoising and enhancement effects for cancer region identification

Spatial heterogeneity in gene expression is a key feature of tumor tissues, crucial for the identification of cancer regions [31]. However, technical noise in sequencing data often obscures key biological signals. We find that by predicting gene expression from histological images, FmH2ST achieves natural denoising and enhancement of raw gene expression. Moreover, it clearly reveals major cancer regions and makes subtle biological signals visible.

**Figure 8.**
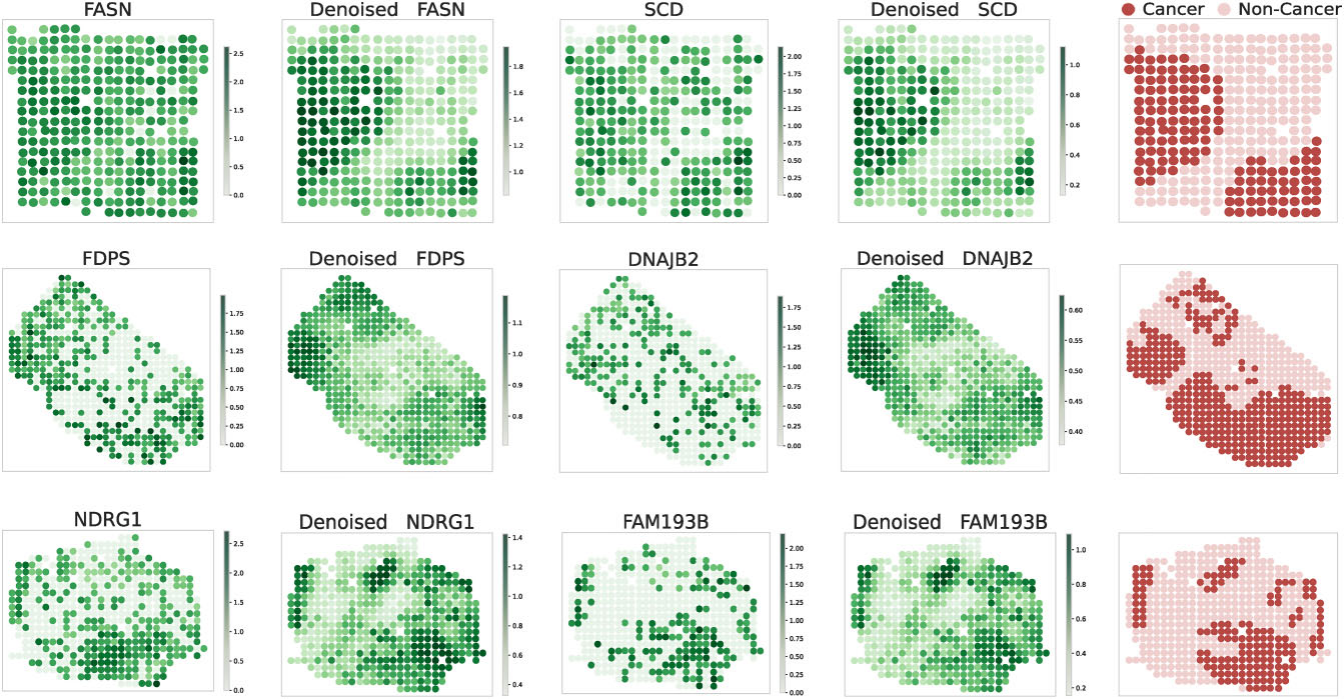
Comparison of raw and FmH2ST’s predicted gene expression patterns across tumor tissue sections. Spatial gene expression patterns are visualized in sections D1 (FASN, SCD), E1 (FDPS, DNAJB2), and G2 (NDRG1, FAM193B). For each gene, the left panel shows the raw gene expression patterns, while the right panel shows denoised ones (predicted gene expression patterns), alongside annotated cancer regions (rightmost panels).

To validate this finding, we conduct experimental analyses on the HER2+ dataset. First, we select some tissue sections with annotated cancer regions and identify differentially expressed genes (using scanpy.tl.rank_genes_groups method) between cancer and noncancer regions within these sections. These differentially expressed genes exhibit more pronounced expression differences between cancer and noncancer regions. We visualize several representative highly differential genes in selected sections (D1, E1, and G2), most of which are closely associated with breast cancer [42–44, 55–57]. For example, SCD shows particularly high expression in HER2+ breast cancer and is closely associated with poor prognosis [42], while NDRG1 is highly expressed in aggressive breast cancer, where it acts as an oncogene promoting tumor growth and metastasis [55, 56]. Further biological analysis for these differentially expressed genes is provided in Supplementary Note 1.5. Figure 8 compares the raw expression patterns of these genes with our predictions, alongside their corresponding cancer region annotations (rightmost panels in red).

Compared to raw gene expression, the predicted expression reveals more clear spatial expression patterns and signal clarity. In the raw data, while these differentially expressed genes show general expression patterns that correspond to cancer regions, the signals are noisy and the boundaries between different regions are less distinct. FmH2ST’s predicted gene expression shows more clear boundaries between cancer and noncancer regions. Specifically, in section D1, two distinct cancer regions become clearly visible, and in section E1, FmH2ST successfully reveals three well-defined cancer regions that are less apparent in the raw data. This enhancement is particularly striking in section G2, where the central cancerous region shows relatively subtle expression differences in the raw data but emerges as a well-defined area with distinctly elevated expression in our predictions.

Overall, these results suggest that the gene expression patterns learned by FmH2ST achieve natural denoising of raw gene expression and significantly improve cancer region identification, which has potential clinical implications for characterizing tumor heterogeneity and treatment strategies.

### FmH2ST better maintains gene co-expression patterns

Considering that noise may obscure genuine gene co-expression relationships and given FmH2ST’s effective denoising capability, we conduct gene co-expression analysis. This analysis examines whether FmH2ST captures complex gene–gene interactions rather than simply matching expression distributions, which is essential for identifying functionally related genes and regulatory networks.

We perform the analysis on the HER2+ dataset. Specifically, we calculate the mean expression value of each gene across all spots within the section and select the top 50 highly expressed genes for subsequent analysis. The selection of highly expressed genes ensures robust correlation analysis and focuses on genes likely to have functional significance. For each tissue section, we then compute PCC between each pair of selected genes, resulting in a 50 × 50 correlation matrix. This analysis is conducted on both the observed gene expression and predictions from different methods (including FmH2ST, THItoGene, Hist2ST, and HisToGene) to compare their performance. We focus our analysis on tissue sections from Patient C (C1–C6), where more pronounced patterns of gene correlation are found in the observed gene expression. The gene–gene correlation matrices from six tissue sections are visualized using heatmaps, with PCC represented by a color spectrum ranging from dark blue to dark red.

**Figure 9.**
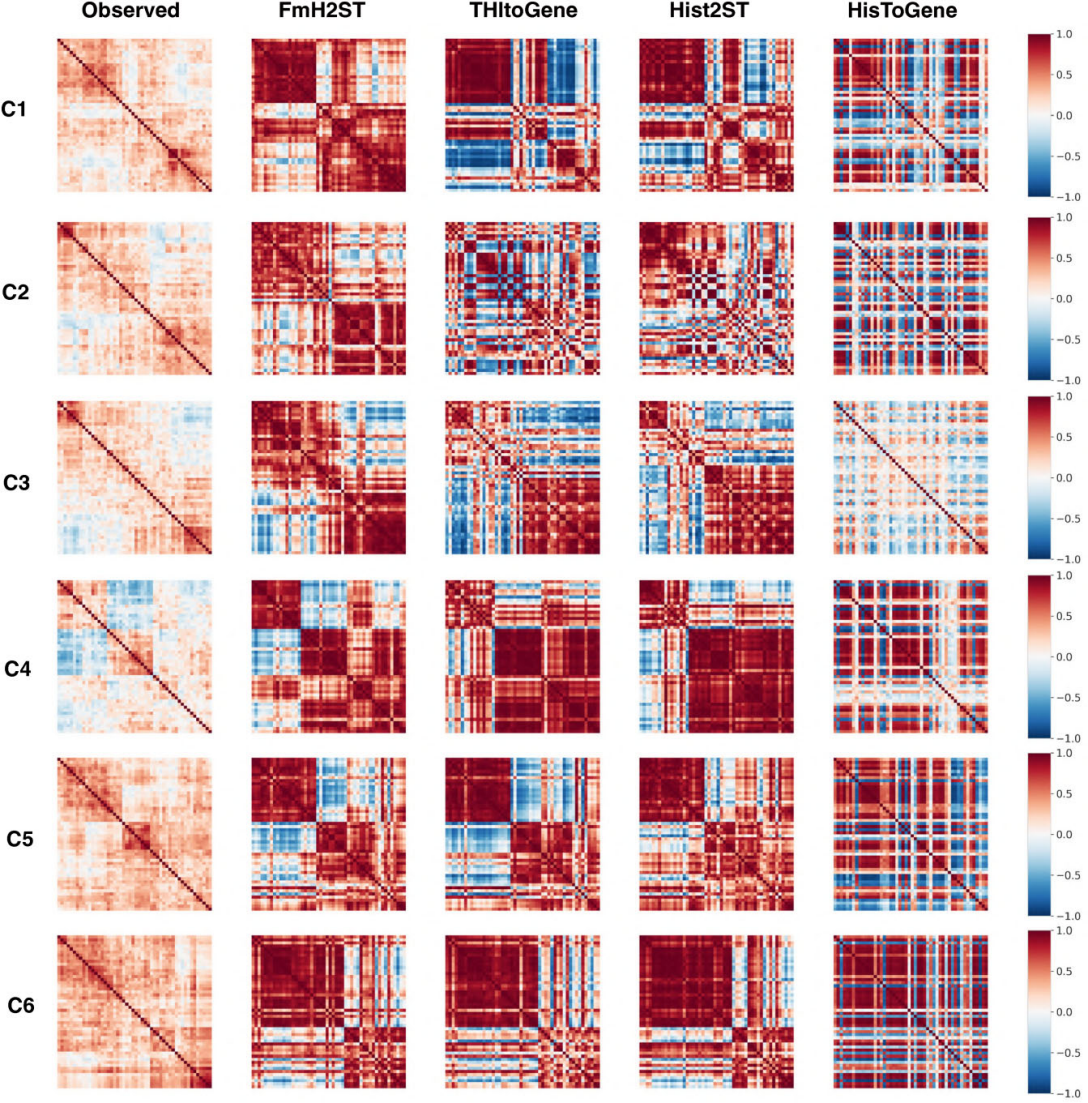
Gene–gene co-expression analysis of different methods across tissue sections. Heatmaps show PCC between the top 50 highly expressed genes, with values ranging from −1 to 1. The analysis compares correlation patterns between observed gene expressions and predictions from FmH2ST, THItoGene, Hist2ST, and HisToGene across six tissue sections (C1–C6) from Patient C in HER2+ dataset.

As shown in Fig. 9, the observed gene expression reveals distinct block structures, indicating gene co-expression relationships. Our correlation analysis demonstrates that FmH2ST achieves better performance in three key aspects: (i) accurate preservation of primary correlation blocks, including both highly correlated regions and uncorrelated or negatively correlated ones; (ii) appropriate enhancement of co-expression pattern visibility; and (iii) sensitive detection of correlation intensity, capturing subtle variations in gene relationships. In contrast, THItoGene and Hist2ST show limited capability in capturing correlation blocks and tend to oversimplify relationship patterns. HisToGene has the worst performance, displaying dispersed association patterns and introducing noisy correlations not present in the observed data, leading to its exclusion from subsequent analyses.

To study these advantages, we conduct a comprehensive comparative analysis across different tissue sections. While these three methods have similar performance in sections C5 and C6, FmH2ST exhibits superior capabilities in other more challenging sections. Notably, in sections C2 and C3, FmH2ST successfully identifies two major positive correlation blocks, while other compared methods fail to maintain co-expression patterns and produce chaotic expression patterns. In section C1, these compared methods tend to produce extreme correlation values, particularly exaggerated negative correlations, which distort the true biological relationships between genes. In contrast, FmH2ST shows appropriate enhancement and preserves more authentic biological relationships. Furthermore, in section C4, it shows enhanced sensitivity in detecting subtle variations within negative correlation blocks, which are missed by the compared methods.

Overall, the gene co-expression analysis indicates that FmH2ST not only better predicts individual gene expression but also maintains meaningful co-expression signals. It can consistently capture the main correlation blocks while preserving subtle variations in correlation patterns, providing a solid foundation for inferring GRNs and pathway interactions.

### FmH2ST improves GRN inference through enhanced gene expression prediction

GRNs are crucial for understanding cellular functions and biological mechanisms, yet their accurate inference remains challenging due to technical noise and data sparsity in sequencing data. Given that gene expression profiles generated by FmH2ST exhibits denoising effects and preserves gene co-expression patterns, we investigate its potential to discover biologically meaningful GRNs.

**Figure 10.**
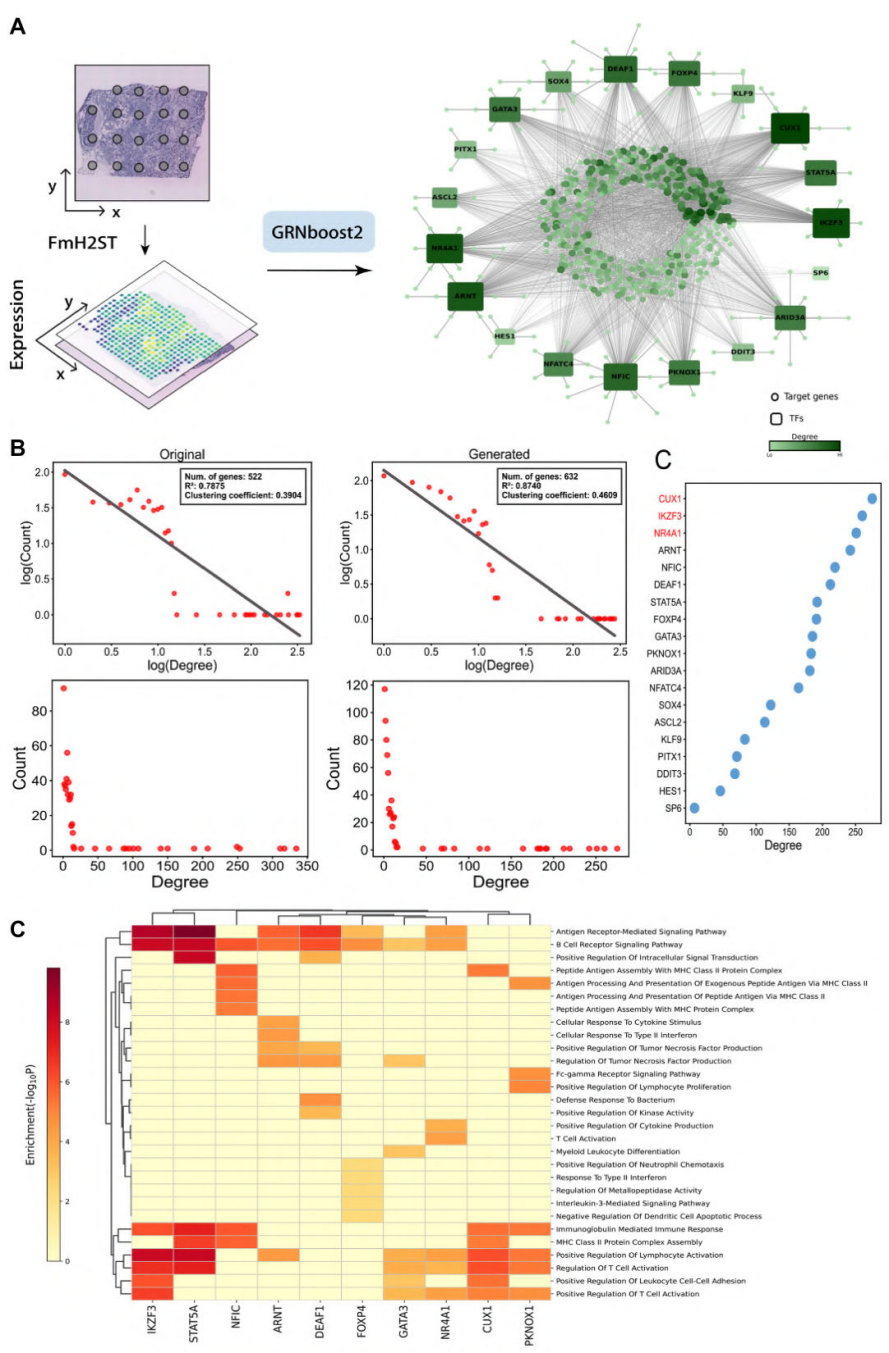
GRN inference from expression data predicted by FmH2ST. (**A**) The pipeline from tissue image to GRN. Left: Tissue image and predicted expression profiles. Right: Inferred GRN where square nodes represent TFs and circular nodes represent target genes. (**B**) The log–log plots of degree distributions for networks from original (left) and predicted (right) gene expression data, with their corresponding degree count plots below. (**C**) Out-degree ranking of TFs in the network, with key regulatory factors highlighted in red. (**D**) Heatmap of GO enrichment analysis results for regulons of the top 10 TFs.

We establish a systematic pipeline to infer GRNs from ST tissue sections. Taking the B1 slice in the HER2+ dataset as an example, we first identify 19 TFs from 785 predicted genes. We then use the GRNBoost2 [58] algorithm for GRN inference. To enhance network reliability, we retain the top 3000 edges based on their confidence scores, resulting in a network comprising 632 nodes. Figure 10A illustrates the computational workflow and the inferred network derived from FmH2ST’s predictions, where square nodes represent TFs and circular nodes represent target genes, with node size and color intensity proportional to node degree. For comparison, we apply the same inference procedure to the original expression data, also retaining the top 3000 edges, which give a network of 521 nodes and allow us to systematically compare GRNs constructed from predicted and original gene expression.

GRNs built from gene expressions predicted by FmH2ST exhibit more desirable biological characteristics than the original ones. As shown in Fig. 10B, both networks display power-law degree distributions, a fundamental characteristic of scale-free networks in biological systems [59], where a few nodes (hubs) have many connections while most nodes have few. We quantify the fit to a power law by applying a logarithmic transformation to the degree distributions and calculating the coefficient of determination (R^2^). The GRN inferred from predicted gene expressions has a higher R^2^ value (0.8740) compared to the network from original data (0.7875), manifesting a better scale-free property of biological networks. Additionally, the clustering coefficient increases from 0.3904 to 0.4669, which is more consistent with the intrinsic characteristics of natural biological networks [59]. These improvements are owing to FmH2ST’s capability to address sparsity and high noise in the original data, enabling the inferred GRN to better capture regulatory relationships.

Network topology analysis of TF outdegree distribution (Fig. 10C) reveals potential key regulatory factors. TFs in the network exhibit distinct hierarchical regulatory characteristics. We focus on the top five TFs, which display high outdegrees and multiple regulatory relationships with target genes, suggesting their potential roles as key master regulators in the network. Among them, CUX1, IKZF3, and NR4A1 (highlighted in red in the figure) are well-documented cancer-associated factors. These three TFs play important roles in breast cancer development and progression: CUX1 is associated with poor prognosis in ER-positive breast cancer [60], NR4A1 promotes tumor cell metastasis [61], and IKZF3 is linked to poor prognosis in HER2+ and regulates cell proliferation [62]. The identification of these critical regulators validates the reliability of our network and provides insights into breast cancer transcriptional regulation.

To explore the biological functions of these key TFs, we perform GO enrichment analysis on the regulons of the top 10 TFs ranked by out-degree (Fig. 10D). The analysis reveals that multiple regulons are indeed closely associated with specific biological functions. For instance, IKZF3, reported to regulate both T-cell activation and B-cell receptor signaling [63, 64], aligns with our GO enrichment analysis results. Furthermore, our enrichment analysis identifies both shared and distinct functional patterns among regulons. For instance, IKZF3 and STAT5A overlap in immune-related pathways [65, 66], while FOXP4 exhibits some distinct enrichment patterns associated with breast cancer [67, 68], suggesting potential cooperative and specialized regulatory mechanisms.

Overall, the regulatory network analysis demonstrates that gene expression profiles predicted by FmH2ST are well-suited for GRN inference, as evidenced by the improved network properties including higher R^2^ values and clustering coefficients. The identification of key regulators of breast cancer and their biologically coherent functional enrichment patterns further validate the reliability of our inferred network. These results highlight the power of FmH2ST on generating ST and its utility for studying tissue-specific gene regulation.

## Discussion

In this study, we present FmH2ST, an economical and efficient alternative for ST data sequencing (Supplementary Note 1.6). FmH2ST leverages a novel dual-branch learning framework that effectively combines prior knowledge from foundation model with fine-grained details from spot images for enhanced spatial gene expression prediction. The dual-branch design of the method exhibits complementarity. As illustrated in Fig. 4A and Supplementary Fig. S4, the foundation model branch achieves higher accuracy in some slices, while the spot-specific feature branch gains better accuracy in others. This indicates that each branch has its distinctive advantages and validates the necessity of fusing features of two branches. For most slices, the performance of fused features surpasses that of either branch alone, but not for a few exceptional slices, suggesting that these slices may have unique tissue structures. Future research could analyze these special slices to further clarify the advantages of each branch and design a more efficient fusion mechanism.

Beyond prediction accuracy, another significant advantage of FmH2ST is its denoising capability. Observed gene expression in ST is often confounded by technical factors including sequencing depth, batch effects, and background noise. We observe that FmH2ST demonstrates notable denoising and enhancement, particularly in visualizing differentially expressed genes in cancer regions, where it dissects more clear cancer spatial heterogeneity. Study has shown that image information contributes to effectively denoising in ST data [69]. Since FmH2ST leverages images for gene expression prediction, we speculate that this image information incorporation may naturally achieve denoising effects. FmH2ST incorporates two types of image features: spot-level local features and slice-level global features. Interestingly, we find that global features appear to play an important role in the denoising process. As shown in Supplementary Fig. S6, when analyzing the predicted gene expression without global features, we observe reduced denoising effects across slices, particularly in slice E1, where the model misses the main cancer regions. We speculate that the importance of global features arises from the heterogeneity across tissue sections, as spots within the same slice likely share similar technical noise patterns. By incorporating global features, the model can better distinguish spots from different slices and mitigate shared technical noise within each slice, thus achieving more effective denoising.

While FmH2ST demonstrates denoising and enhancement effects in gene expression prediction, an important concern is whether these effects might eliminate crucial biological signals. Our gene co-expression analysis addresses this concern by revealing that FmH2ST’s predictions faithfully preserve meaningful biological relationships present in the observed data, confirming that the method achieves effective denoising while maintaining essential biological information rather than simply smoothing the data.

Furthermore, in GRNs, we observe that the GRN constructed from FmH2ST’s predictions demonstrate enhanced biological properties, including more pronounced power–law distribution and higher clustering coefficients. We analyze that these improvements in network properties may benefit from two key factors of FmH2ST: effective denoising and well-preserved gene co-expression patterns. Sequencing-based gene expression data is often impacted by technical noise and data sparsity, where technical noise can lead to false correlations and data sparsity may mask true regulatory relationships. In our study, the predicted gene expression demonstrates natural denoising and enhancement while maintaining gene co-expression patterns, which may explain why we can construct regulatory networks with stronger biological characteristics. These results support the biological reliability of FmH2ST’s predictions and highlight the potential of FmH2ST in denoising, signal-preserving, and improving GRN inference.

Although FmH2ST achieves competitive performance, it still has some limitations. First, its reliance on the quality and compatibility of the chosen foundation model may affect performance, suggesting the importance of model selection. Second, the time complexity of FmH2ST’s dual-branch framework is slightly higher than that of single-branch methods. Addressing these limitations in future work could further expand the model’s applicability.

In summary, our framework provides a learning paradigm that simultaneously leverages priors from the foundation model branch and fine-grained details from spot images. Additionally, the modular design of our framework allows for easy extension to other foundation models, enabling the potential utilization of more powerful pre-trained foundation models. With the advancement of sequencing technologies, more sophisticated ST platforms such as HDST [70] can provide single-cell resolution, while stereo-seq platforms [71] can even measure gene expression at subcellular resolution. Our dual-branch framework is well-suited for these high-resolution datasets, connecting histological images and gene expression at finer resolutions, providing new opportunities for image-based analysis of cancer region distribution, cell–cell interactions, and gene regulatory mechanisms at single-cell resolution.

## Conclusion

In this study, we present FmH2ST, a foundation model-based method for spatial gene expression prediction. FmH2ST is designed to overcome three key challenges: (i) the lack of external prior knowledge and insufficiency in capturing (ii) inter-slice heterogeneity and (iii) complex intra-slice relationships. To achieve this, FmH2ST (i) employs a dual-branch architecture to integrate prior knowledge from foundation model and fine-grained details from spot images, (ii) models inter-slice heterogeneity through multilevel feature extraction strategy, leveraging both spot-level and slice-level image features to enhance spot features with slice context, and (iii) introduces a dual-graph strategy that augments the canonically used spatial graph with a learnable feature graph to capture semantic relationships between similar but spatially distant spots.

Compared to existing methods, FmH2ST performs better in gene expression prediction. Notably, the predicted gene expression demonstrates effective denoising and enhancement, enabling a clearer revelation of cancer regions while preserving critical gene co-expression relationships. Based on these characteristics, its application to GRN inference yields more biologically meaningful results and enables identification of key regulators. These capabilities, combined with its cost-effectiveness, position FmH2ST as a potential tool for clinical diagnostics and research.

## Supplementary Material

gkaf865_Supplemental_File

## Data Availability

The human HER2-positive breast tumor spatial transcriptomics dataset used in this paper is available at https://github.com/almaan/her2st/. The human cutaneous squamous cell carcinoma 10× Visium data is available at GSE144240 (https://www.ncbi.nlm.nih.gov/geo/query/acc.cgi?acc=GSE144240). FmH2ST is implemented in Python. Its source code is available at https://www.sdu-idea.cn/codes.php?name=FmH2ST.
