# Supplementary material for: FmH2ST: foundation model-based spatial transcriptomics generation from histological images
Source: Nucleic Acids Res. 2025 Sep 9;53(17):gkaf865. doi: 10.1093/nar/gkaf865 (PMC12418390; doi:10.1093/nar/gkaf865)
Supplement: gkaf865_Supplemental_File [file gkaf865_supplemental_file.pdf]

Supplementary Information:

FmH2ST: Foundation model-based spatial transcriptomics generation from histological images

Yuequn Wang[a,b], Jun Wang[b], Yanyu Xu[b], Ning Liu[a], Bin Liu[c], Yuliang Li[c,*], and Guoxian Yu[a,b,*]

a. School of Software, Shandong University, Jinan, 250101, Shandong, China.

b. SDU-NTU Centre for Artificial Intelligence Research, Shandong University, Jinan, 250101, China.

c. Department of Interventional Medicine and Minimally Invasive Oncology, Shandong University, Jinan, 250033, China.

This document contains:

-Supplementary Note 1.1 to 1.6.

-Supplementary Figures S1 to S6.

-Supplementary Tables S1 to S2.

1 Supplementary Note

1.1 Hyperparameter k_f analysis for feature graph

In Section 2.3.2 of the main text, we constructed the feature graph by selecting $k_f = 8$ as the number of neighbors in the KNN algorithm. To evaluate the sensitivity of the model to the choice of k_f, we conducted the following experiment. We set k_f to $\{4, 8, 10, 12\}$, respectively, and performed a comprehensive evaluation on the slice samples from 7 patients in the HER2+ dataset. Specifically, we extracted the first slice from each patient (samples "A2", "B1", "C1", "D1", "E1", "F1", and "G1"). For each slice, we calculated the mean Pearson correlation coefficient (Mean PCC), median Pearson correlation coefficient (Median PCC), and mean squared error (MSE) of the prediction results under different k_f values, and averaged the results of the 7 slices to obtain an overall evaluation of the model performance under different k_f values. The experimental results show that the model exhibits some robustness to the selected k_f values and is not particularly sensitive. As shown in Supplementary Figure 1 A and B, both Mean PCC and Median PCC reach their highest values when $k_f = 8$. As shown in Supplementary Figure 1 C, the mean squared error (MSE) reaches its lowest value when $k_f = 8$. Overall, the model performs best when $k_f = 8$ within the tested range of k_f values. This indicates that selecting $k_f = 8$ can effectively avoid introducing excessive noise while capturing non-local dependencies, thereby achieving better prediction performance. Therefore, we chose $k_f = 8$ as the default value for feature graph construction in the experiment.

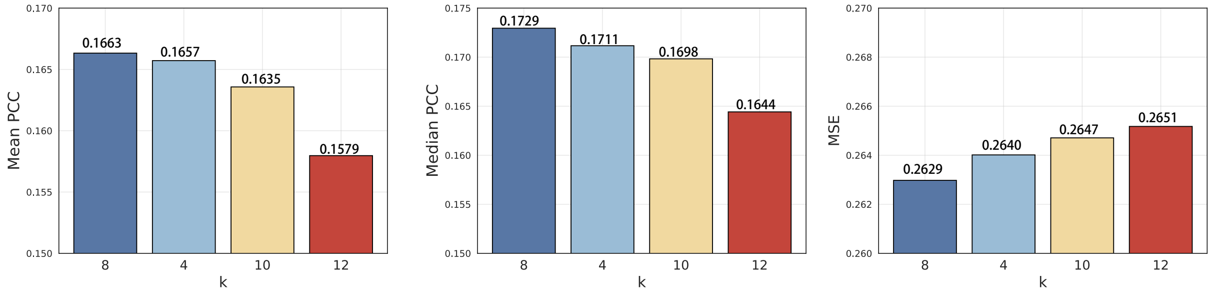

Supplementary Figure 1: Sensitivity analysis of different k_f values in Feature Graph Construction. (A) Mean PCC, (B) Median PCC, and (C) MSE averaged across 7 patient slices for different k_f values $\{4, 8, 10, 12\}$.

1.2 Analysis of different kernel sizes

To select suitable kernel sizes for multi-scale convolutions, we refer to previous studies [1, 2] and evaluate three sets of kernel sizes: {3, 5, 7}, {7, 9, 11}, and {9, 11, 13}. As shown in Supplementary Figure 2, these three kernel size sets achieve comparable performance. Larger kernel sizes show a slight performance decrease but remain effective. The performance across kernel size sets demonstrates that FmH2ST is robust to kernel size selection. Since larger kernel sizes offer no further improvements but increase computational cost, we therefore adopt {3, 5, 7} as the kernel sizes in the model.

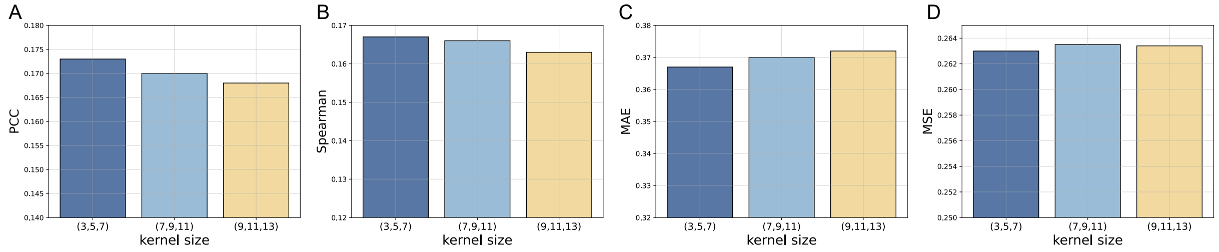

Supplementary Figure 2: Comparison of different convolution kernel size combinations on model performance, evaluated by (A) PCC, (B) Spearman, (C) MAE, and (D) MSE.

1.3 Analysis of multi-component loss function

To evaluate both the computational and predictive impact of incorporating the three independent MLPs and the multi-component loss in FmH2ST, we analyze model complexity and compare the complete FmH2ST model with a simplified variant, in which the outputs of the two branches are directly fused for prediction, without the two branch MLPs and their corresponding loss terms.

The comparison of model parameters for the complete FmH2ST and the simplified variant is summarized in Supplementary Table 1. Since the two branch MLPs are implemented as single-layer networks, the increase of parameters is minimal (1.41% for HER2+ and 0.31% for cSCC), without introducing significant additional complexity to the model.

To evaluate the impact of the two branch MLPs, we compare the mean PCC and MSE results of the complete FmH2ST and its simplified variant on representative sections from the HER2+ dataset (the first section from each patient) and the cSCC dataset. As shown in Supplementary Tables 2 and 3, FmH2ST achieves higher PCC and lower MSE in most

sections, indicating the effectiveness of the multi-component loss without introducing notable additional complexity.

Supplementary Table 1: Comparison of model parameters with and without the two branch MLPs, and the increased parameters

**Dataset**	**w/o branch MLPs**	**FmH2ST**	**ΔParams (%)**
HER2+	57.36M	58.17M	1.41%
cSCC	56.46M	56.63M	0.31%

Supplementary Table 2: Comparison of PCC and MSE values between models with and without the two branch MLPs in HER2+ dataset

**Slice**	**PCC**		**MSE ↓**	
	**w/o branch MLPs**	**FmH2ST**	**w/o branch MLPs**	**FmH2ST**
A2	**0.110**	0.106	0.337	**0.336**
B1	0.296	**0.312**	0.286	**0.280**
C1	0.239	**0.256**	0.224	**0.221**
D1	**0.183**	0.175	**0.248**	0.253
E1	0.091	**0.106**	**0.180**	0.182
F1	**0.086**	0.073	**0.289**	0.290
G1	0.160	**0.180**	0.284	**0.280**

Supplementary Table 3: Comparison of PCC and MSE values between models with and without the two branch MLPs in cSCC dataset

**Slice**	**PCC**		**MSE ↓**	
	**w/o branch MLPs**	**FmH2ST**	**w/o branch MLPs**	**FmH2ST**
P2_ST_rep1	0.333	**0.337**	**0.619**	0.667
P2_ST_rep2	**0.322**	0.301	**0.579**	0.611
P2_ST_rep3	**0.387**	0.381	**0.561**	0.582
P5_ST_rep1	0.091	**0.099**	0.623	**0.598**
P5_ST_rep2	0.084	**0.114**	0.630	**0.611**
P5_ST_rep3	0.108	**0.112**	0.635	**0.612**
P9_ST_rep1	0.237	**0.241**	0.742	**0.741**
P9_ST_rep2	0.246	**0.248**	0.737	**0.727**
P9_ST_rep3	0.186	**0.193**	**0.662**	0.667
P10_ST_rep1	0.175	**0.207**	0.659	**0.633**
P10_ST_rep2	0.207	**0.209**	0.579	**0.578**
P10_ST_rep3	0.174	**0.203**	0.567	**0.559**

1.4 Performance analysis of foundation models in FmH2ST

To compare the impact of different foundation models on the performance of FmH2ST, we integrate PLIP [3], Prov-GigaPath [4], and PathoDuet [5] into the FmH2ST framework under the same experimental configuration. The foundation models differ in pretraining strategies and feature extraction capabilities. PLIP is a multimodal model that leverages vision-language alignment during pretraining to enhance the extraction of semantically relevant features from pathology images. Prov-GigaPath and PathoDuet are both ViT-based models, with Prov-GigaPath utilizing the DINOv2 self-supervised training [6] on approximately 300 million pathology image patches, while PathoDuet adopts a cross-scale localization pretraining strategy to associate features at different magnifications, thereby facilitating comprehensive multi-scale tissue characterization.

For the comparative experiments, we select the first slice from each patient (A2, B1, C1, D1, E1, F1, and G1) in the HER2+ dataset and evaluate the mean values of four metrics (PCC, Spearman, MSE, and MAE) for each foundation model, as shown in Supplementary Table 4. The result shows that PathoDuet achieves slightly better performance across all metrics, and Prov-GigaPath ranks second. This supports the choice of PathoDuet as the backbone model, likely due to its cross-scale feature learning strategy. Furthermore, these results indicate that the FmH2ST framework is compatible and extensible with different foundation models.

Supplementary Table 4: Performance comparison of different foundation models on the HER2+ dataset.

**Model**	**PCC**	**Spearman**	**MSE ↓**	**MAE ↓**
PLIP	0.166	0.161	0.276	0.381
Prov-GigaPath	0.171	0.164	0.266	0.371
PathoDuet	**0.173**	**0.167**	**0.263**	**0.367**

1.5 Detailed biological analysis of differentially expressed genes

Most of the differentially expressed genes between cancer and non-cancer regions are closely associated with the development and progression of breast cancer, participating in various biological processes. **SCD** promotes tumor cell proliferation and survival by regulating the fatty acid desaturation process. SCD is highly expressed in HER2-positive breast cancer and is closely associated with aggressive tumor characteristics. Moreover, elevated SCD

expression positively correlates with the level of immune cell infiltration within the tumor immune microenvironment, suggesting that SCD may indirectly influence the immune status of tumors [7]. Similarly, high expression of **FASN** is associated with rapid tumor proliferation and increased aggressiveness. FASN supplies cancer cells with fatty acids necessary for energy production and membrane synthesis, thereby supporting tumor growth and progression [8]; inhibition of FASN can suppress cancer cell proliferation and metastasis [9]. High expression of **NDRG1** in aggressive breast cancers, such as triple-negative breast cancer, significantly enhances the migratory and invasive abilities of tumor cells and promotes the occurrence of brain metastases. In addition, NDRG1 facilitates tumor cell growth and survival by activating the AKT-mTOR signaling pathway and regulating iron metabolism. In clinical samples, elevated NDRG1 expression is closely associated with shorter overall survival and breast cancer-specific survival, and serves as an independent prognostic factor [10, 11]. **FDPS** is a critical enzyme in the cholesterol biosynthesis pathway and has been closely linked to the development and progression of breast cancer. Enhanced FDPS activity promotes elevated intracellular cholesterol levels, which drive the proliferation, migration, and invasion of breast cancer cells. Moreover, FDPS can modulate the functions of immune cells within the tumor microenvironment, thereby promoting tumor growth, metastasis, and immune evasion. FDPS is also considered as a potential therapeutic target for breast cancer [12].

Although DNAJB2 and FAM193B have not yet been conclusively linked to breast cancer initiation or progression, there are indications of their potential involvement. For **DNAJB2**, it is noteworthy that members of the heat shock protein family, including the Hsp40/DNAJ family, are commonly overexpressed in various tumor tissues, including breast cancer. These proteins help cancer cells maintain protein homeostasis, resist apoptosis, and adapt to adverse microenvironments [13]. As such, the differential expression of DNAJB2 in breast cancer tissues may represent an adaptive mechanism by which tumor cells cope with stress and promote survival. As for **FAM193B**, although it has not been extensively studied in breast cancer, it has demonstrated oncogenic activity in other cancers, such as clear cell renal cell carcinoma, where it drives tumor proliferation through the PI3K/AKT and MAPK/ERK signaling pathways [14]. Notably, the PI3K/AKT signaling pathway is widely activated in breast cancer (approximately 60% breast cancers have hyperactivation of this pathway) and

plays a key role in the proliferation, survival, and metabolic reprogramming of tumor cells [15, 16]. Given that oncogenic mechanisms tend to be shared across cancer types [17], it is plausible that FAM193B may also exhibit similar pro-tumorigenic functions in breast cancer. These observations provide new clues for exploring the potential roles of DNAJB2 and FAM193B in breast cancer.

1.6 Time and cost analysis of FmH2ST and spatial transcriptomics sequencing

To assess the practical efficiency of FmH2ST compared to spatial transcriptomics sequencing, we evaluated both the time and cost required for each approach. In terms of time, taking the HER2-positive breast cancer dataset used in our study as an example, training the FmH2ST model typically requires about 5–6 hours on an NVIDIA A100 GPU, or about 7–8 hours on an RTX 3090. Once the model is trained, inference on a new H&E-stained tissue section can be completed within seconds to a few minutes. In contrast, the spatial transcriptomics sequencing workflow (e.g., 10x Visium) involves multiple steps, including tissue collection, fixation, H&E staining, permeabilization, cDNA synthesis, tissue removal, cDNA release, library construction and sequencing [18]. The entire process from sectioning to sequencing typically takes 2–4 days, excluding instrument queue and data analysis time [18]. Notably, the preparation of H&E-stained slices is necessary for both approaches, but this step is relatively fast (usually 1–2 hours) [18] and routine in clinical workflows.

In terms of cost, spatial transcriptomics sequencing requires substantial expenditure on reagents, specialized chips, sequencing resources, and skilled personnel. For example, on the 10x Visium platform, the cost of library preparation per tissue section is approximately 2,800–4,600 USD (according to UCSF Genomics CoLab public pricing), with sequencing costs charged separately according to data volume. In comparison, FmH2ST only requires computational resources for training, without complex wet-lab experimental procedures or manual intervention, resulting in a much lower overall cost (less than 25 USD for training, based on Google Cloud A100 GPU pricing). Once trained, inference on new H&E-stained tissue sections incurs almost no additional cost and requires only minimal computational resources.

Overall, FmH2ST provides an efficient and economical alternative to spatial transcriptomics sequencing, enabling rapid and economical spatial gene expression analysis from rou-

tinely available histological images.

2 Supplementary Figures

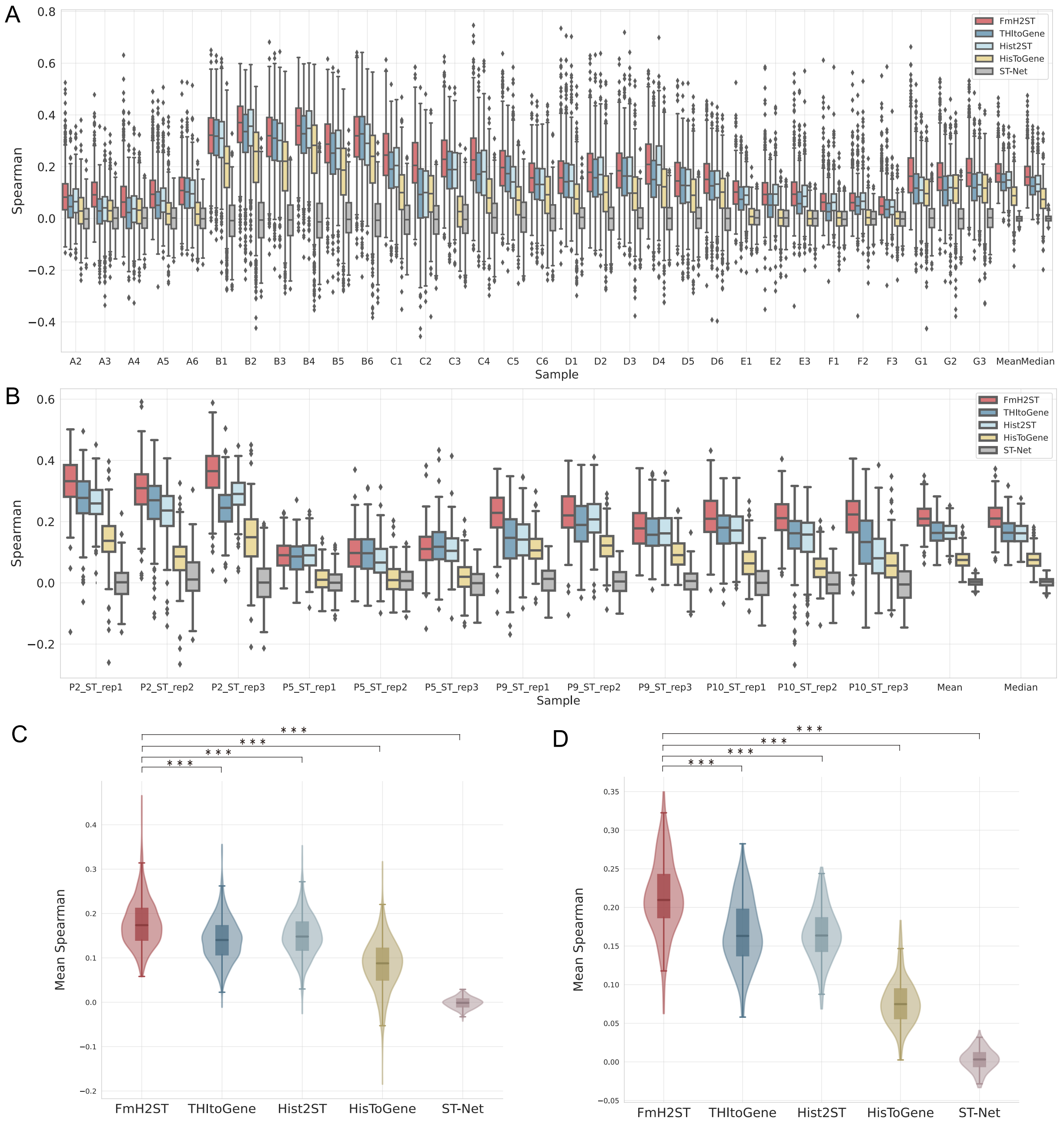

Supplementary Figure S1: Comparison of prediction performance of different methods. (A-B) Box plots showing the distribution of Spearman's Rank Correlation Coefficient for different methods across tissue sections in (A) HER2+ dataset (32 sections) and (B) cSCC dataset (12 sections). (C-D) Violin plots with statistical comparison (Wilcoxon signed-rank test) of mean Spearman values across all cross-validation experiments for (C) HER2+ dataset and (D) cSCC dataset. *** indicates p-value < 0.001.

A

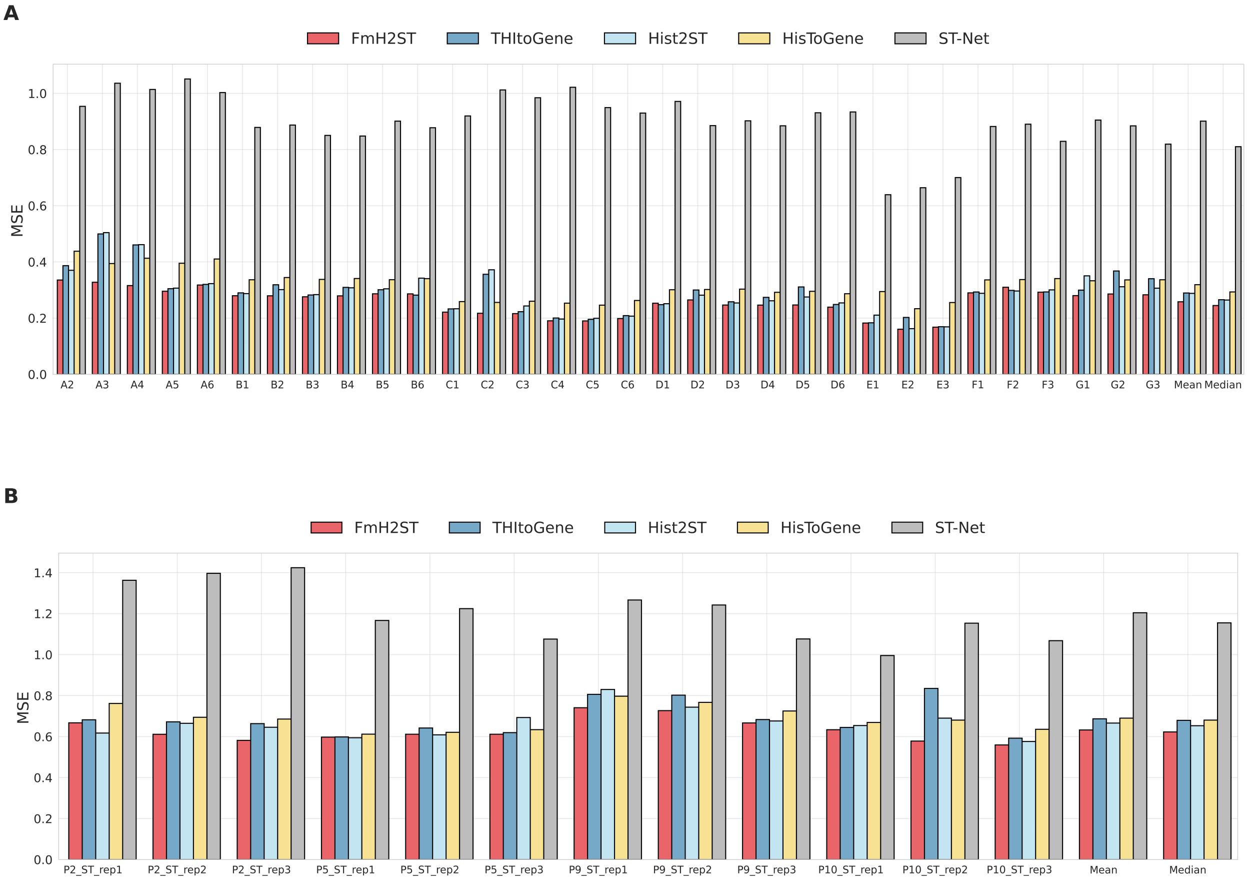

Supplementary Figure S2: Comparison of MSE across different methods. (A-B) Bar plots showing the mean squared error (MSE) of different methods in (A) HER2+ dataset (32 tissue sections) and (B) cSCC dataset (12 tissue sections). Lower bars indicate better prediction performance.

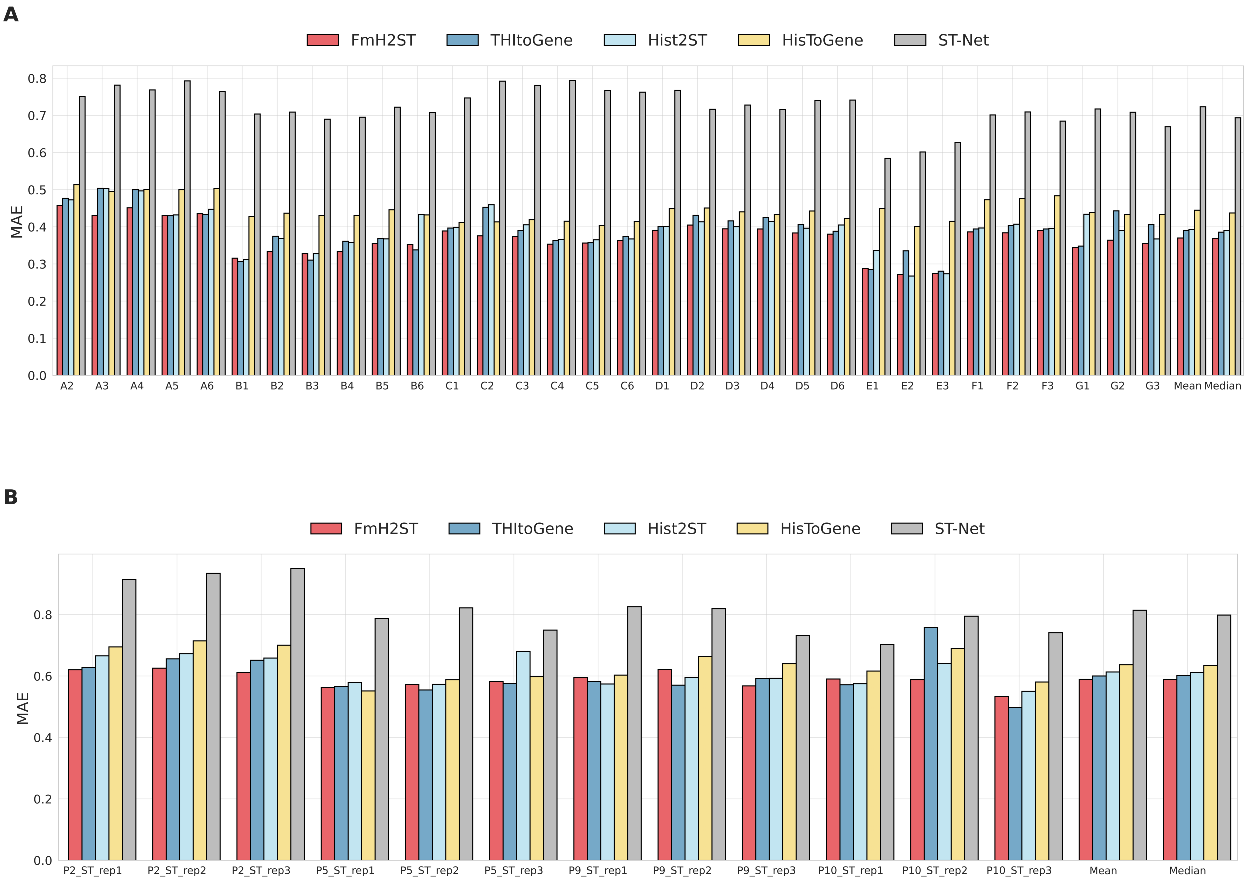

Supplementary Figure S3: Comparison of MAE across different methods. (A-B) Bar plots showing the mean absolute error (MAE) of different methods in (A) HER2+ dataset (32 tissue sections) and (B) cSCC dataset (12 tissue sections). Lower bars indicate better prediction performance.

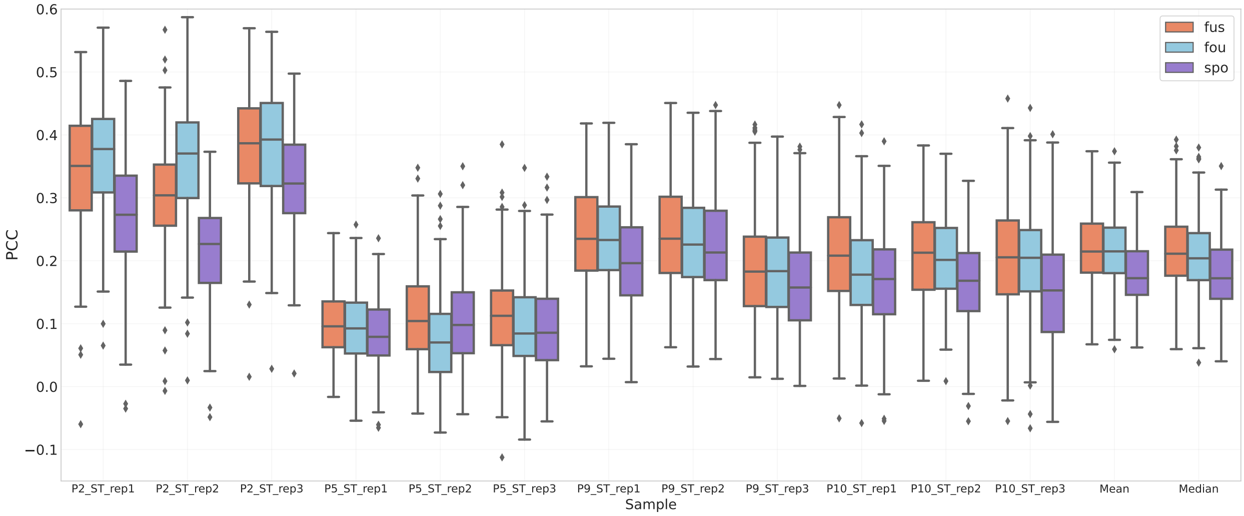

Supplementary Figure S4: Performance comparison of different branches in the dual-branch framework across tissue sections in the cSCC dataset. The boxplot shows the PCC between predicted and original gene expression for each tissue section using three different prediction branches: fusion branch (fus), foundation model branch (fon), and spot-specific branch (spo).

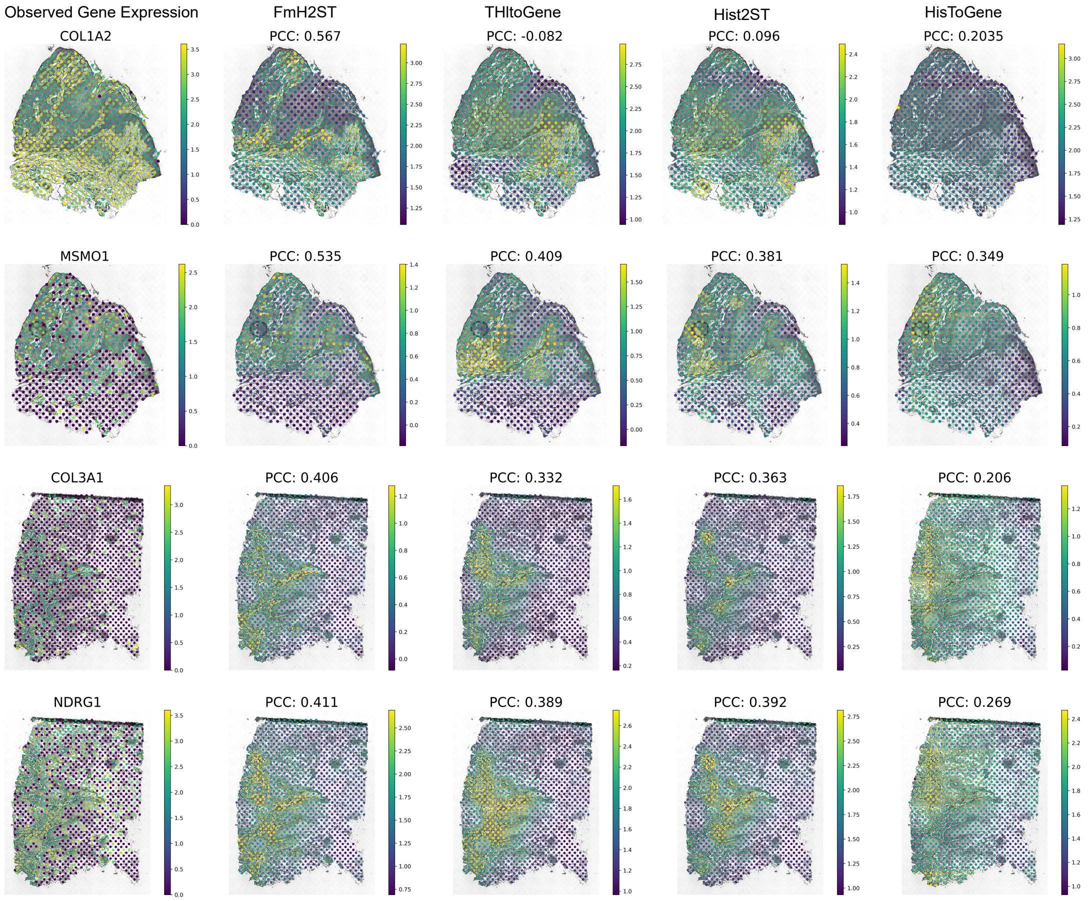

Supplementary Figure S5: Visualization of the predicted four genes with the highest mean -log10 p-values across 12 tissue sections are shown. P-values were calculated from correlations between predicted and observed gene expressions. For each gene, the tissue section with the lowest p-value is selected for visualization. Spots with darker coloring indicate lower expression levels, while lighter coloring represents higher expression levels.

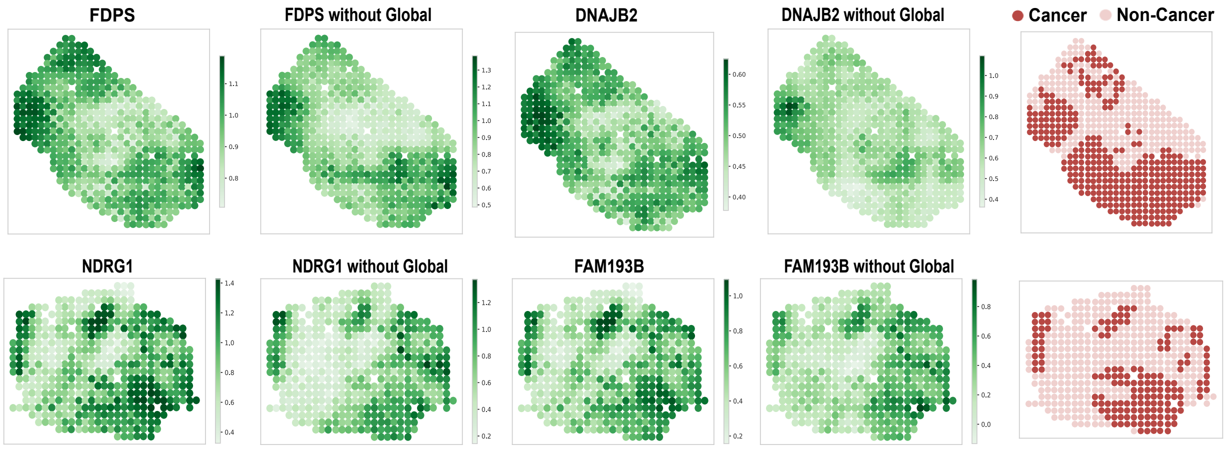

Supplementary Figure S6: Comparison of gene expression predictions with and without global image features across tumor tissue sections. Spatial gene expression patterns visualized in tissue sections E1 (FDPS, DNAJB2), and G2 (NDRG1, FAM193B). For each gene, the left panel shows predictions using the full model (with global features), while the right panel shows predictions without global features, alongside annotated cancer regions (rightmost panels, where red indicates cancer regions and white indicates non-cancer regions). Expression levels are represented by green intensity, with darker green indicating higher expression.

3 Supplementary Tables

Supplementary Table S1: The top 50 genes ranked by their mean -log10 p-values across all tissue sections in the HER2+ dataset. The p-values were calculated from the correlation between predicted and observed gene expression patterns for each tissue section. Lower p-values (higher -log10 p-values) indicate stronger statistical evidence against the null hypothesis of no correlation between predicted and observed expression patterns.

Rank	Gene	Mean -log10 p-values	Rank	Gene	Mean -log10 p-values
1	FN1	20.57902312	26	GPRC5A	10.27265774
2	GNAS	18.87919669	27	TMBIM6	10.01585183
3	SCD	18.22109053	28	MID1IP1	9.91548993
4	FASN	17.66843206	29	COL3A1	9.86400618
5	IGKC	17.03824764	30	TMEM14B	9.81475136
6	IGHA1	15.95595182	31	IGLC3	9.63392745
7	MYL12B	14.18147631	32	VIM	9.61568562
8	CLDN4	13.79115725	33	KRT8	9.60114244
9	TMEM123	13.18581469	34	PDCD5	9.57561473
10	STMN1	13.01042754	35	HLA-B	9.56166456
11	RHOB	12.70198732	36	SRGN	9.53232707
12	ITGB6	12.63440438	37	NDRG1	9.42350891
13	MUCL1	12.31331533	38	NDUFB3	9.39197652
14	HLA-DRA	12.01148714	39	TNC	9.27226686
15	IGLC2	11.89337280	40	SPARC	9.15717013
16	C3	11.75096402	41	POSTN	9.00681455
17	CCT4	11.73609048	42	GATA3	8.96675017
18	HMGB2	11.47760852	43	NDUFA1	8.83173069
19	IGHG3	11.06390756	44	MRPL51	8.82617991
20	TXNDC17	11.06057840	45	HSPB1	8.73628581
21	CD74	10.98185998	46	TANC2	8.69711916
22	CRABP2	10.73163069	47	HLA-DPB1	8.68254094
23	SRSF1	10.71752236	48	ARF6	8.61425175
24	NDUFB2	10.66060792	49	HLA-DRB1	8.59481973
25	LUM	10.27776566	50	CRACR2B	8.55474352

Supplementary Table S2: The top 50 genes ranked by their mean -log10 *p*-values across all tissue sections in the cSCC dataset. The *p*-values were calculated from the correlation between predicted and observed gene expression patterns for each tissue section. Lower *p*-values (higher -log10 *p*-values) indicate stronger statistical evidence against the null hypothesis of no correlation between predicted and observed expression patterns.

Rank	Gene	Mean -log10 *p*-values	Rank	Gene	Mean -log10 *p*-values
1	COL1A2	28.03034965	26	PTHLH	18.55740371
2	MSMO1	27.50985418	27	PTP4A2	18.17116415
3	COL3A1	23.89930894	28	CALML5	17.88822815
4	NDRG1	23.57279736	29	SPRR2D	17.66312061
5	SBSN	23.21902361	30	IGFL1	17.61396685
6	FDFT1	22.54535989	31	F3	17.32856250
7	HSPH1	22.01038963	32	CTNND1	16.83087963
8	ITGA6	21.69393344	33	7-Mar	16.58896121
9	DMKN	21.28710902	34	CYFIP1	16.40624959
10	NHP2	21.12655850	35	PSMA7	15.97995130
11	EFNB1	21.04718592	36	IMP4	15.19616892
12	MOB1A	20.90783613	37	KLF6	14.65700459
13	TMOD3	20.41735576	38	SRP72	14.55893207
14	NEFL	20.15116347	39	WNK1	14.39787607
15	PI3	20.08159688	40	PACS1	14.35412729
16	SPINK5	19.86442914	41	CSNK1A1	14.13798089
17	STMN1	19.85231328	42	HSP90AA1	14.13626763
18	CASP14	19.23325865	43	ZFP36L2	14.09104554
19	KRTDAP	19.22460495	44	NAP1L1	14.04514791
20	PAICS	19.10785791	45	EIF5	13.98679917
21	RPL13	19.08063010	46	MTDH	13.93966104
22	ENAH	19.07636227	47	SMC4	13.91969115
23	DIAPH1	18.97320760	48	CASP4	13.91956813
24	PRDX4	18.83223611	49	KCTD1	13.81137367
25	RALA	18.56784776	50	MAST4	13.62712287

References

[1] Feng Chen, Jiangshu Wei, Bing Xue, and Mengjie Zhang. Feature fusion and kernel selective in inception-v4 network. *Applied Soft Computing*, 119:108582, 2022.

[2] Hafiz Tayyab Mustafa, Jie Yang, and Masoumeh Zareapoor. Multi-scale convolutional neural network for multi-focus image fusion. *Image and Vision Computing*, 85:26–35, 2019.

[3] Zhi Huang, Federico Bianchi, Mert Yuksekgonul, Thomas J Montine, and James Zou. A visual–language foundation model for pathology image analysis using medical twitter. *Nature Medicine*, 29(9):2307–2316, 2023.

[4] Hanwen Xu, Naoto Usuyama, Jaspreet Bagga, Sheng Zhang, Rajesh Rao, Tristan Naumann, Cliff Wong, Zelalem Gero, Javier González, Yu Gu, et al. A whole-slide foundation model for digital pathology from real-world data. *Nature*, 630(8015):181–188, 2024.

[5] Shengyi Hua, Fang Yan, Tianle Shen, Lei Ma, and Xiaofan Zhang. Pathoduet: Foundation models for pathological slide analysis of h&e and ihc stains. *Medical Image Analysis*, 97:103289, 2024.

[6] Maxime Oquab, Timothée Darcet, Théo Moutakanni, Huy Vo, Marc Szafraniec, Vasil Khalidov, Pierre Fernandez, Daniel Haziza, Francisco Massa, Alaaeldin El-Nouby, et al. Dinov2: Learning robust visual features without supervision. *arXiv preprint arXiv:2304.07193*, 2023.

[7] Jie Wang, Qian Zhang, Duanrui Zhou, Yixuan Wang, Huilian Che, Yunjun Ge, Zhangfeng Zhong, and Guosheng Wu. Systematic analysis of fatty acid desaturases in breast invasive carcinoma: The prognosis, gene mutation, and tumor immune microenvironment. *Medicine*, 103(25):e38597, 2024.

[8] Chiara Papulino, Ugo Chianese, Ahmad Ali, Gregorio Favale, Concetta Tuccillo, Fortunato Ciardiello, Annabella Di Mauro, Chiara Mignogna, Gerardo Ferrara, Alfredo Budillon, et al. Inverse fasn and ldha correlation drives metabolic resistance in breast cancer. 22(1):676, 2024.

[9] Barbara Schroeder, Travis Vander Steen, Ingrid Espinoza, Chandra M Kurapaty Venkatapoorna, Zeng Hu, Fernando Martín Silva, Kevin Regan, Elisabet Cuyàs, X Wei Meng, Sara Verdura, et al. Fatty acid synthase (fasn) regulates the mitochondrial priming of cancer cells. *Cell Death & Disease*, 12(11):977, 2021.

[10] Emilly S Villodre, Xiaoding Hu, Bedrich L Eckhardt, Richard Larson, Lei Huo, Ester C Yoon, Yun Gong, Juhee Song, Shuying Liu, Naoto T Ueno, et al. Ndrg1 in aggressive breast cancer progression and brain metastasis. *Journal of the National Cancer Institute*, 114(4):579–591, 2022.

[11] Sukanya B Jadhav, Michaela Vondrackova, Petra Potomova, Cristian Sandoval-Acuña, Jana Smigova, Kristyna Klanicova, Daniel Rosel, Jan Brabek, Jan Stursa, Lukas Werner, et al. Ndrg1 acts as an oncogene in triple-negative breast cancer and its loss sensitizes cells to mitochondrial iron chelation. *Frontiers in Pharmacology*, 15:1422369, 2024.

[12] Jia Lu, Siwei Chen, Xuejiao Bai, Minru Liao, Yuling Qiu, Ling-Li Zheng, and Haiyang Yu. Targeting cholesterol metabolism in cancer: From molecular mechanisms to therapeutic implications. *Biochemical Pharmacology*, 218:115907, 2023.

[13] Daniel R Ciocca and Stuart K Calderwood. Heat shock proteins in cancer: diagnostic, prognostic, predictive, and treatment implications. *Cell Stress & Chaperones*, 10(2):86, 2005.

[14] Guohai Xie, Xinyi Zheng, Zhong Zheng, Ruoyu Wu, Zhixian Yao, Wenjie Huang, Feng Sun, Xingyu Mu, Ke Wu, and Junhua Zheng. The cerna pvt1 inhibits proliferation of ccrcc cells by sponging mir-328-3p to elevate fam193b expression. *Aging (Albany NY)*, 13(17):21712, 2021.

[15] Miguel A Ortega, Oscar Fraile-Martínez, Ángel Asúnsolo, Julia Buján, Natalio García-Honduvilla, and Santiago Coca. Signal transduction pathways in breast cancer: the important role of pi3k/akt/mtor. *Journal of Oncology*, 2020(1):9258396, 2020.

[16] Ricardo LB Costa, Hyo Sook Han, and William J Gradishar. Targeting the pi3k/akt/mtor pathway in triple-negative breast cancer: a review. *Breast Cancer Research and Treatment*, 169:397–406, 2018.

[17] Bert Vogelstein and Kenneth W Kinzler. Cancer genes and the pathways they control. *Nature Medicine*, 10(8):789–799, 2004.

[18] Alma Andersson, Ludvig Larsson, Linnea Stenbeck, Fredrik Salmén, Anna Ehinger, Sunny Z Wu, Ghamdan Al-Eryani, Daniel Roden, Alex Swarbrick, Åke Borg, et al. Spatial deconvolution of her2-positive breast cancer delineates tumor-associated cell type interactions. *Nature Communications*, 12(1):6012, 2021.
